# Sequence Profiling of the *Saccharomyces cerevisiae* Genome Permits Deconvolution of Unique and Multialigned Reads for Variant Detection

**DOI:** 10.1534/g3.113.009464

**Published:** 2014-02-20

**Authors:** Claire Jubin, Alexandre Serero, Sophie Loeillet, Emmanuel Barillot, Alain Nicolas

**Affiliations:** * Institut Curie Centre de Recherche, UMR3244 CNRS, Université Pierre et Marie Curie, 75248 Paris Cedex 05; † Institut Curie Centre de Recherche, INSERM U900, 75248 Paris, France. Ecole des Mines Paris Tech, 77300 Fontainebleau, France

**Keywords:** analysis of repetitive regions, reference sequence bias, genome HTS annotation

## Abstract

Advances in high-throughput sequencing (HTS) technologies have accelerated our knowledge of genomes in hundreds of organisms, but the presence of repetitions found in every genome raises challenges to unambiguously map short reads. In particular, short polymorphic reads that are multialigned hinder our capacity to detect mutations. Here, we present two complementary bioinformatics strategies to perform more robust analyses of genome content and sequencing data, validated by use of the *Saccharomyces cerevisiae* fully sequenced genome. First, we created an annotated HTS profile for the reference genome, based on the production of virtual HTS reads. Using variable read lengths and different numbers of mismatches, we found that 35 nt-reads, with a maximum of 6 mismatches, targets 89.5% of the genome to unique (U) regions. Longer reads consisting of 50−100 nt provided little additional benefits on the U regions extent. Second, to analyze the remaining multialigned (M) regions, we identified the intragenomic single-nucleotide variants and thus defined the unique (M_U_) and multialigned (M_M_) subregions, as exemplified for the polymorphic copies of the six flocculation genes and the 50 Ty retrotransposons. As a resource, the coordinates of the U and M regions of the yeast genome have been added to the *Saccharomyces* Genome Database (www.yeastgenome.org). The benefit of this advanced method of genome annotation was confirmed by our ability to identify acquired single nucleotide polymorphisms in the U and M regions of an experimentally sequenced variant wild-type yeast strain.

The efficiency of DNA sequencing has tremendously increased in recent years, due to the advances of high-throughput sequencing (HTS) technologies and new instruments that allow rapid retrieval of sequence information from total genomes, including the human genome, in an appropriately equipped research laboratory setting. Massive parallel short-read sequencing technologies produce a large collection of individual reads. Afterward, the extraction of information is usually performed by analyzing the results of reads that are aligned against a reference sequence. The success of read mapping greatly depends on the proximity, accuracy, and degree of assembly of the reference genome to the experimental sample and is inevitably filled with noise due to the presence, in every genome, of repetitive regions. Repeated sequences lead to the production of identical or slightly polymorphic reads that originate from different chromosomal locations. Robust analyses therefore depend on how efficiently one can minimize the confusing effects that multialignments pose.

The most intuitive strategy is based on the selection of the “best” alignment among multialignments according to the number of mismatches. Short Oligonucleotide Alignment Program (SOAP) (Li *et al.* 2008b) and Program to Align Short Sequences (PASS) (Campagna *et al.* 2009) programs implement such strategies in which the “best” alignment is defined as the one with the smallest number of mismatches. A second approach is implemented in a program MApping short reads and calling variants using mapping Quality score MAQ (Li *et al.* 2008a) uses the notion of “mappability.” Each alignment is associated with a score, reflecting the probability of correctly placed reads. This information is then used to filter alignments before the search of variants. The third approach, developed here, is a reverse strategy that consists of an advanced preanalysis of the reference sequence. It aims to identify, at a nucleotide-level resolution, the unique and multialigned subgenomic regions. This is achieved by conducting a simulated HTS experiment. Virtual reads are generated along the whole genome and then are back aligned onto this sequence. This is the strategy initiated by Koehler *et al.* (2011) to analyze the unique subpart of a genome where reads are uniquely aligned, while the rest of the genome remains unexplored.

The difficulty in exploring regions containing multi-aligned reads depends on several factors. Biologically, it depends on the global complexity of the genome sequence and the density of repeated motifs, their size, and degree of polymorphisms. Technically, mapping also depends on the accuracy of sequencing, the length of the reads, the number of mismatches allowed during the read mapping step, and in the case of repeated regions, on their polymorphisms. Short reads also decrease the power of assembly for building accurate contigs because repetitive regions create ambiguities (Treangen and Salzberg 2012). Altogether, these factors affect the robustness of the alignment information and lead to potential false-positive (FP) calls in resequencing experiments that aim at identifying genome variants. Thus, in many HTS studies, multialigned reads are set aside, if not completely discarded. For example, in the whole-genome resequencing study of the 1000 Genomes project (Clarke *et al.* 2012), the variant calling was restricted to 80–85% of the human sequence. Referred to as the “accessible genome,” this is defined as the portion of the reference sequence that remains after excluding regions containing ambiguously placed reads or unexpectedly low/high read coverage. More recently, Lee and Schatz (2012) used the expression “dark matter” when referring to multialignment regions that are difficult to analyze. The efforts to identify the “dark matter” consisted of setting-up a new parameter referred to as, the Genome Mappability Score (GMS). GMS is a statistical measure for the probability that a position is at risk of being multialigned, based on read mapping. This parameter gives a score at each position of the genome that can be used to filter the experimental results, according to a threshold that defines the single nucleotide variants (SNVs) termed robustness. Operationally, it reduces FP calls but finally restricts the analysis to highly “mappable” regions.

Here, we report two generic and complementary bioinformatics strategies based on advanced annotations of the reference genome. As a proof of concept, it allowed us to (i) deconvolute the *Saccharomyces cerevisiae* genome into unique (U) and multialigned (M) regions using virtual read analyses, (ii) establish a list of the intragenomic SNVs that characterize whole-genome repetitiveness (provided as a resource in the Saccharomyces Genome Database [SGD] database; http://www.yeastgenome.org/), and (iii) explore the M regions to define subsets of unique regions (M_U_) and identify M region distinguishing variants. The alignment filter we developed, “g-deNoise,” anticipates the impact of intragenomic SNVs in multialignments. These advances allowed us to accurately identify acquired SNVs in both U and M regions of an experimentally sequenced variant yeast strain.

## Materials and Methods

### Production of virtual reads

To generate virtual HTS short reads, the complete genome of *S. cerevisiae* was extracted from the SGD, version February 2011. The reference sequence comes from the S288c strain background, which is a standard genetic background for functional studies (Mewes *et al.* 1997). To match our experimental HTS reads, the reference sequence was first converted into the dinucleotide color space, specific to SOLiD sequencing (Life Technologies), with help of a color to base-space conversion matrix (Breu 2008). Virtually perfect reads of different lengths were generated on the forward strand at each position of all chromosomes analyzed. Virtually perfect 50-nt base-space reads on the forward strand were generated from the same reference.

### Reads mapping

The simulated dinucleotide color space reads were back aligned against the *S. cerevisiae* reference genome using the “mapreads” program from Applied Biosystems SOLiD “corona lite 4.2.2” software package. Our standard mapping parameters were set to allow six mismatches per 50 nt-read alignment. To generate an exhaustive mapping result, no threshold was fixed to limit the number of reported alignments per read. It is noteworthy that six mismatches in color-space are not equivalent to six mismatches in base-space (Breu 2008). In the base-space simulation experiments, the virtual reads were aligned on the reference sequence with the software BFAST (Homer *et al.* 2009). Only alignments without gaps and containing at most three base mismatches were considered. No threshold was fixed to limit the number of alignments reported per read. The draft sequence of the strains BY4741 (BY4741_Toronto_2012) and BY4742 (BY4742_Toronto_2012) (http://www.yeastgenome.org/) also were used to identify constitutive Single Nucleotide Polymorphisms (SNPs) of our laboratory BY4741 and BY4742 strains and were compared with our mutation accumulation lines in the reference S288c sequence.

The experimental 50 nt-reads were aligned on the *S. cerevisiae* reference sequence using the same program, mapreads, and the same parameters were used as for simulated 50 nt-reads: six mismatches were allowed and no threshold was fixed on the number of reported alignments.

### Annotation of U *vs.* M regions

After the mapping step, the alignment coverage plot was computed on each chromosome of the reference genome. These graphs help to visually assess the coverage pattern of the virtual HTS profile. A read is considered to be multialigned if it has more than one alignment. Identification of M regions is performed after discarding the unique alignments from total alignments. Thus, the M regions correspond to genomic regions still covered by these “ambiguous” alignments, whereas the U regions are defined as the complementary regions located between two consecutive M regions. The coordinates of the U and M regions were defined at the level of single-nucleotide resolution, using the S288C reference genome. M region identification was performed for 25-, 35-, 50-, 75-, and 100-bp reads, and for a maximal number of allowed mismatches equaling zero to six. The coordinates of the M regions are available on SGD for 50 and 100 nt-reads and a maximal number of mismatches equal to six (File S1).

### Identification of intragenomic SNVs

Because the virtual HTS reads were generated without introducing nucleotide variations, the presence of mismatched alignments within the M regions only reflects SNVs that distinguish intragenomic repeated sequences. Their coordinates were determined using AnnotateChanges (v0.2, corona-1.0.1r0-4) provided by ABI (Life Technologies). Intragenomic SNVs identified with 50 and 100-nt virtual reads are provided in File S1.

### Yeast strains sequencing

Starting from our frozen *S. cerevisiae* BY4741 strain (Brachmann *et al.* 1998) cell stock (−80° in glycerol), we constructed mutation accumulation lines. Four independent yeast colonies were picked and grown in parallel on YPD (rich medium) plates at 30° upon 100 single cell to colony bottlenecks, generating at the end, four independent clonal lines, wt_A100, wt_B100, wt_C100, and wt_E100. Barcoded and paired-end libraries (50+35 nt), containing fragments of approximately 200 bp, were prepared according to Applied Biosystems (Life Technologies) standard protocols and sequenced on the SOLiD v4 instrument at the HTS platform at the Institut Curie. The sequenced reads we used contain more than 80% of bases with a percentage of sequencing errors less than 0.1%. After mapping with mapreads, from the Applied Biosystems SOLiD corona_lite 4.2.2 software package, the total number of paired-end reads uniquely aligned in U regions was: wt_A100 (15,780,119), wt_B100 (15,438,542), wt_C100 (16,614,434), and wt_E100 (19,658,287), corresponding to a 130- to 200-fold coverage.

### Detection of acquired mutation and biological validation

To identify acquired mutations in the four wild-type experimental samples, SNP identification was first performed with Bioscope (V1.3) in the herein defined U regions. A SNP call was retained if the base change was seen in at least 70% of unique alignments and supported on both strands. Bioscope filters are not adapted to SNP analysis in repetitive regions. Thus, in M regions, we first performed base change calls starting from color mismatches with AnnotateChanges, then we filtered multialignments with the new “g-deNoise” filtering method reported here. At last we extracted acquired SNPs supported by alignments that became uniquely aligned after filtering. A call was validated if it was detected by 10 unique alignments on each strand corresponding at least to five different starting points. “Noisy” homopolymers and microsatellite tracts were eliminated. The parameters were set such that any FP was obtained after Sanger sequencing confirmation. To align the reads, we used the S288C version June 2008 (S288C_reference_genome_R61-1-1_20080605), and the results were converted into coordinates consistent with the version from February 2011.

To validate calls for SNP variation between the reference genome and the sequenced strains, the corresponding regions were PCR amplified and Sanger sequenced (Big Dye Terminator V1.1 Cycle sequencing kit from Life Technologies) using standard conditions. Results from these four wild-type strains were also used to compare the mutational landscape of wild-type and mutator strains (Serero *et al.* 2014).

## Results

**Figure 1:**
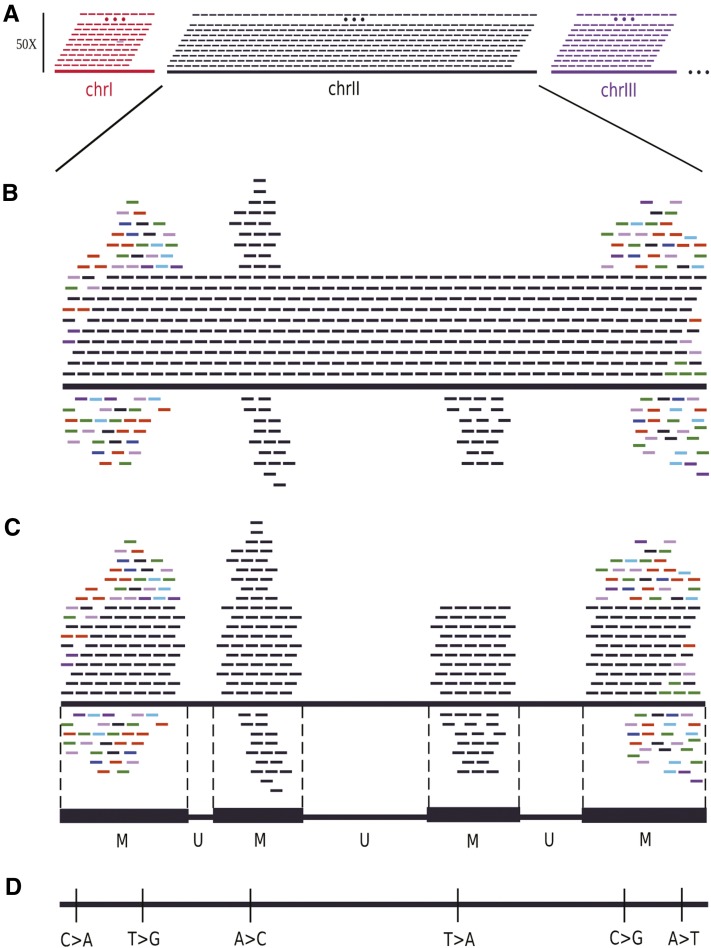
Principle of virtual HTS profiling of a reference genome. (A) Virtual read production. A read is generated at each genomic position on the forward strand so that the coverage reaches the read length (50× in this example). The next steps are illustrated with the chromosome II. (B) Read mapping. Although reads were only generated on the forward strand, some of them, coming from repetitive regions within the same chromosome (black reads) or different chromosomes (colored reads) exhibit multialignments and/or are aligned on the reverse strand. (C) Annotation of the M and U regions. The start and end coordinates of continuous M regions are reported after unique alignments were discarded. Gaps between M regions are converted to U regions. (D) Intragenomic SNV identification. The mismatches analysis in M regions allowed identification of the indicated SNVs found in repetitive regions of the genome. HTS, high-throughput sequencing; SNV, single-nucleotide variant.

To generate a virtual HTS profile from a reference genome, our strategy as illustrated in Figure 1 was the following. Stepwise, we: (i) broke down the reference genome sequence into virtual short reads; (ii) aligned these reads back onto the reference genome, using the same parameters as used for the experimental HTS data; (iii) identified the chromosomal coordinates of the unique (U) and multialigned (M) regions; and (iv) annotated the SNV variants that internally distinguish the M regions.

### Mapping of the U and M regions

Virtual reads of various lengths (25, 35, 50, 75, and 100 nt) were generated at each nucleotide position of the yeast reference genome on the forward strand (Figure 1A). Afterward, as illustrated in Figure 1B, the virtual reads were back aligned against the reference genome with a given number of allowed mismatches. Thus, the final coverage, or full coverage, depends on the read length. For example, virtual read generation for a 50 nt-read length produced a 50-fold coverage. After mapping, the uniquely aligned U regions exhibited the expected 50-fold coverage, whereas some regions exhibited a variable excess of coverage, reflecting multi-aligned M regions. Although the virtual reads were generated on the forward strand, reads were occasionally aligned on the reverse strand, indicating multialigned sequences on opposite strands (Figure 1B). The start and end coordinates of the continuous genomic regions, covered by multi-alignments, define the M regions (Figure 1C). The sum of the U regions defines the *S. cerevisiae* U genome, or “uniqueome” as termed by Koehler *et al.* (2011), and complementarily, the sum of M regions defines the *S. cerevisiae* M genome, or “dark matter” as termed by Lee and Schatz (2012), thus providing an operational annotation for HTS experiments and a readout of the genome sequence architecture and its complexity. The M regions are visualized as peaks with different heights, meaning that reads, generated in other regions of the genome, are cross-aligned. The annotation of these peaks is described below. The annotated details of the M regions within the *S. cerevisiae* genome, based on the virtual HTS profile, are available at SGD. The visualization of M regions in the SGD browser is illustrated in Supporting Information, Figure S1.

### Annotation of the M regions according to the read length and mismatch tolerance

**Figure 2:**
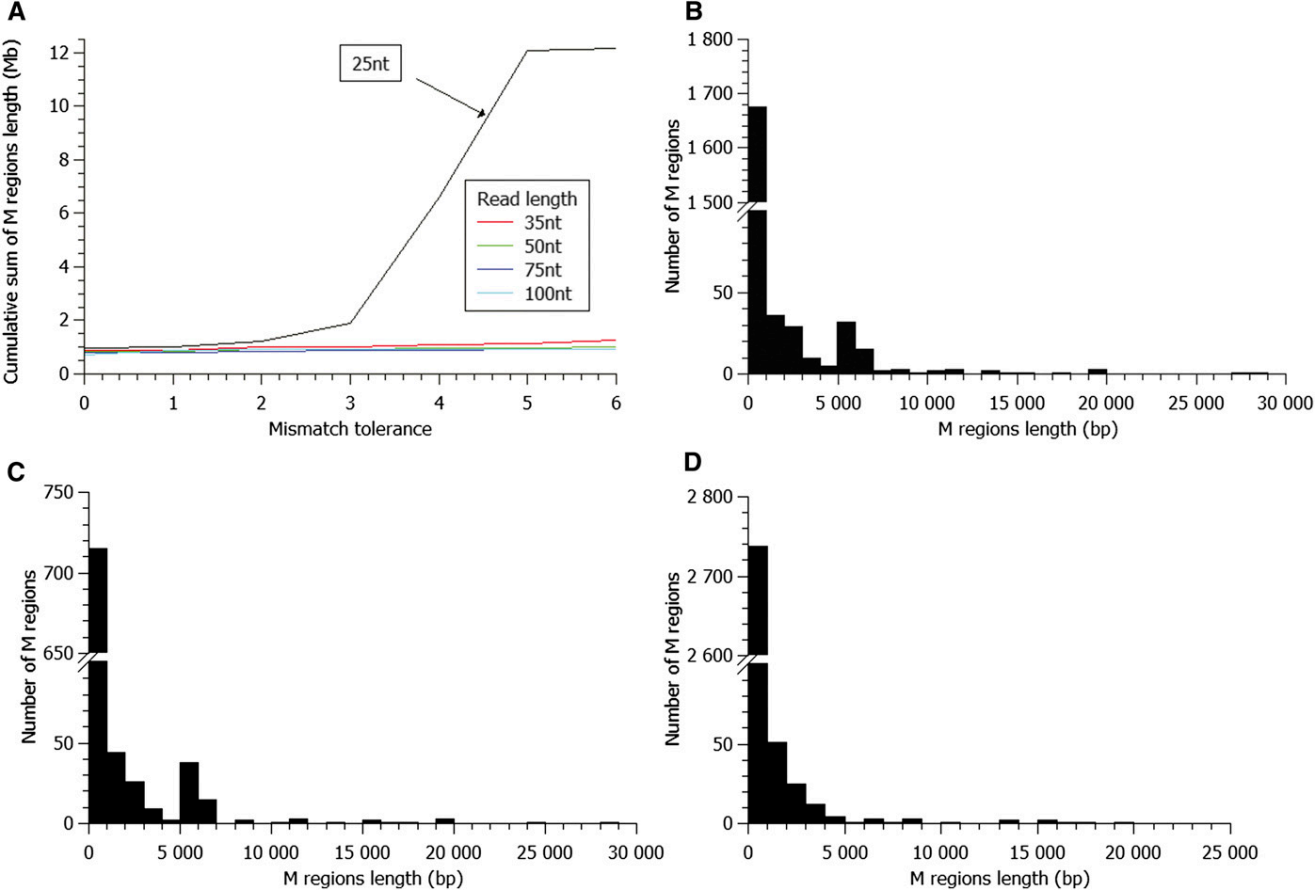
Characterization of M regions. (A) Cumulative length of M regions according to the read length and the number of tolerated mismatches. Histogram of M region lengths annotated with (B) 50nt color space reads with six mismatches allowed, (C) 100nt color space reads with six mismatches allowed, and (D) 50nt base space reads with three mismatches allowed.

**Figure 3:**
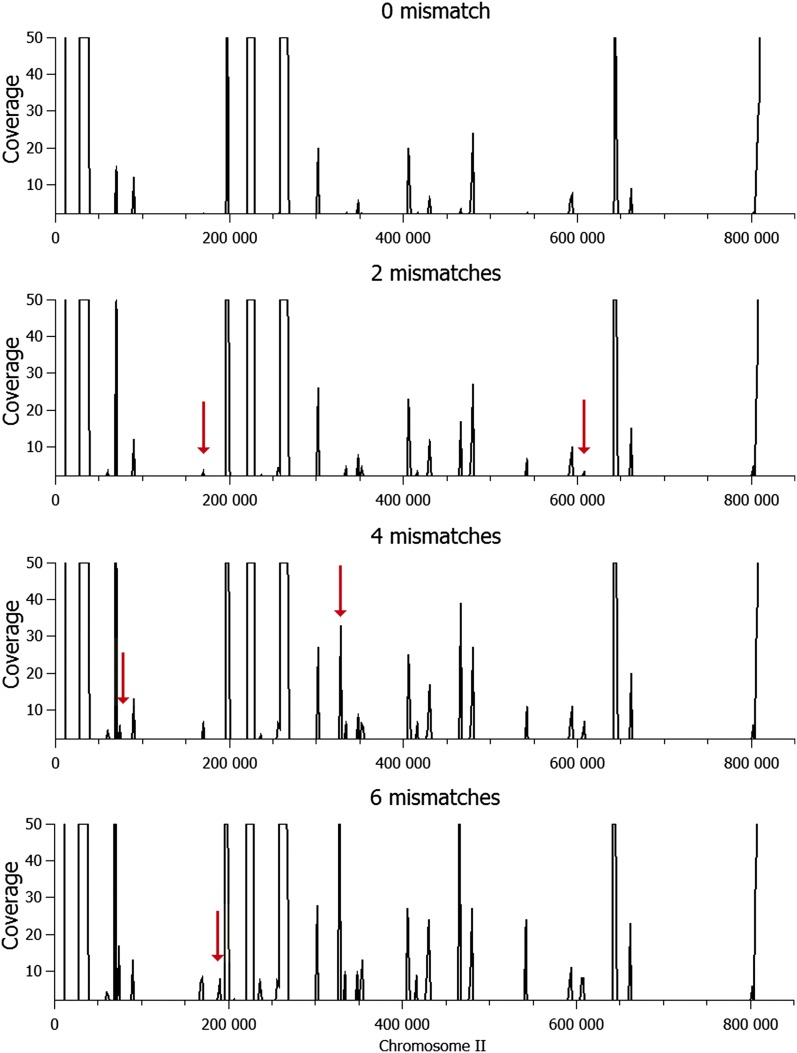
50 nt-read multialignment coverage on the *S. cerevisiae* chromosome II for 0, 2, 4, and 6 allowed mismatches. Peaks greater than 50 are cut off.

Varying the read lengths and the tolerance for mismatches is expected to influence the extent of multialignments during the mapping step. Therefore, we examined the impact of these parameters for virtual read lengths of 25, 35, 50, 75, and 100 nucleotides, while varying the maximal number of mismatches permitted. The results are reported in Figure 2A. As we see for 25 nt-reads, the cumulative sum of M regions rapidly increases from three to six mismatches so that the whole-genome sequence becomes fully covered by M regions. In contrast, for read lengths ≥35 nt, the cumulative length of M regions is much smaller (approximately 10% of the genome) and is weakly sensitive to the number of mismatches permitted. As an example, the impact of mismatch tolerance is illustrated in Figure 3 for chromosome II. This result shows that increasing the number of mismatches leads to the appearance of additional and specific peaks of increased coverage with variable heights. For low coverage peaks, we observe that there are a few isolated multialigned positions, corresponding to low noise motif redundancies, a feature that is expected to increase with enhanced mismatch tolerances per read. Indeed, increasing the number of mismatches from zero to six leads to few additional peaks of low coverage, but roughly similar M profiles are observed. Quantitatively, if one now varies the length of the reads with a maximum tolerance of six mismatches, the percentage of the yeast genome covered by M regions only slightly varies from 10.5% (35 nt-reads) to 8.4% (50 nt-reads), down to 7.7% with 100 nt-reads. When read length increases, the total number of M regions in the genome regularly decreases from 5765 (35 nt), 1824 (50 nt), 1148 (75 nt), and 873 (100 nt) (Figures 2, B and C). Altogether, these analyses indicate that in terms of repetitiveness within the *S. cerevisiae* genome, there is a marked loss of genome complexity when one considers polymorphic sequence motifs shorter than 35 nt-reads. However, it is expected that this threshold of genome complexity will vary from one organism to another, depending on the overall base composition (GC percent), strand biases and the genome content in terms of repetitiveness and evolved history, for example if derived from an ancestral genomic duplication.

### Robustness of the M regions according to the method of virtual read production

To compare reads generated with different sequencing technologies, we also performed the HTS simulation by generating virtual reads in base space (for example as in Illumina sequencing) rather than color space (like in SOLiD sequencing). Using fixed parameters, 50 nt-reads and a maximal number of three mismatches in base space (equivalent to six mismatches in color space), the percentage of genome covered by M regions, after mapping with the BFAST software, was slightly lower than in color space (6.55% *vs.* 8.4%). The M regions identified in base space simulation are included in the M regions identified in color space. The additional M-specific regions identified with color space simulation are due to the enhanced calls in low complexity sequences and exquisitely discriminate the Ty regions (see peaks at 5−6 kb in Figure 2B compared with Figure 2D). Overall, the identification of the M regions is robust and is only marginally sensitive to the method of virtual read-encoding and alignment strategies.

### Comparison between simulated and experimental U and M regions

**Figure 4:**
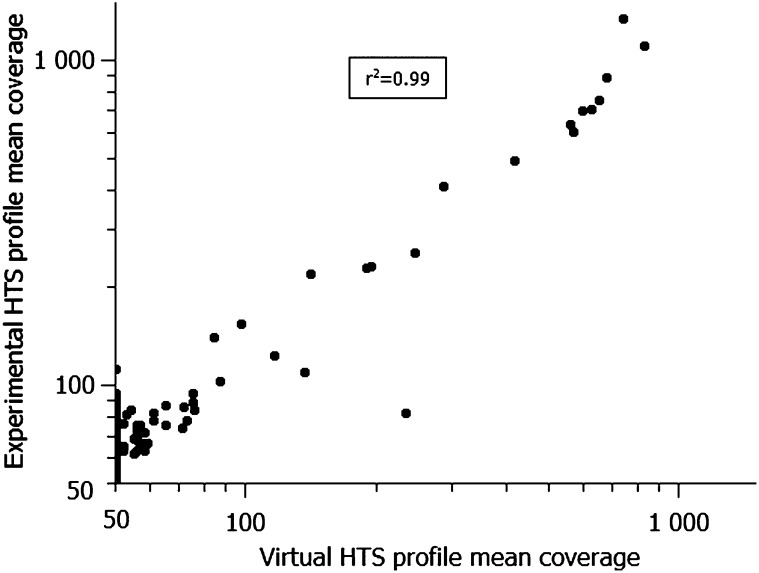
Correlation of coverage between the virtual HTS profile and experimental HTS profile from the *S. cerevisiae* chromosome II. Each point corresponds to the mean coverage of chromosome positions (2-kb window), in Log10 scale, computed before any alignment filtering (U + M regions), on virtual HTS profile (x-axis) and on experimental HTS (y-axis). Coverage is computed for 50 nt-reads and six mismatches. HTS, high-throughput sequencing.

To validate the accuracy of our virtual HTS results, we performed HTS of the wild-type BY4741 strain (Brachmann *et al.* 1998), a strain closely related to the S288c *S. cerevisiae* reference sequence (Mewes *et al.* 1997). As detailed in the section *Materials and Methods*, four independent yeast lines, subjected to 100 single-cell colony bottlenecks (wt_A100, wt_B100, wt_C100, and wt_E100) were paired-ended sequenced (50+35 nt). The alignment of the experimental 50 nt-reads was set to tolerate a maximum of six color-space mismatches. Then, the read coverage was calculated using the set of U regions defined by our virtual HTS profile and corresponding to 91.6% of the genome. The comparison of the U and M regions of the *S. cerevisiae* genome, defined by the virtual HTS procedure, and the experimental data, is illustrated in Figure 4. The correlation between these two sets of data are very strong (r^2^ = 0.99), indicating that the preannotation of the reference genome is extremely accurate to help the analysis of experimental samples.

### Deeper annotation of the M regions

To develop a bioinformatics strategy, to analyze repetitive DNA, we first examined the number and length distribution of the M regions, as defined in the virtual sequencing using 50 nt-reads and a maximum of six mismatches per read. The M regions exhibit large length variations (Figure 2B). The longest M region (28,934 nt) corresponds to a region containing a Ty (yeast retrotransposon) and the rDNA clusters (only one cluster is annotated in the reference genome) on chromosome XII, whereas many M regions exhibit much shorter lengths with a minimal length of 50 nt, limited by the read length resolution. In comparison, the longest U region measures 107,899 nt (144,151−252,049 on chromosome XIV) and can be as short as one nucleotide when it separates two flanking M regions, for example at position 699,128 on chromosome XV.

The M regions with larger lengths and higher coverage correspond to known repetitive elements within the yeast genome (Figure S2, Table S1). This includes the Ty families, the associated and solo long terminal repeats, transfer RNA genes, and telomeric regions. Included are the duplicated genes encoding the H3 and H4 histones on chromosomes II and XIV, and other dispersed or clustered multigene families such as the *FLO* genes, involved in cell flocculation. In other organisms, as in *S. cerevisiae*, multialigned reads from various sources are expected to occur, but with higher frequencies and in larger sized genomes and/or genomes with marked differences in sequence complexity (GC content or presence of pseudogenes and low complexity motifs).

Despite the fact that virtual reads were generated on the forward strand, certain reads aligned on the reverse strand. As an example, Figure S3 reports the forward and reverse coverage on chromosome II. Alignments on the reverse strand illustrate that some reads are generated within inverted repeat motifs elsewhere, in the genome. Effects of multialignments, due to duplication, also are observed within the histone *HHF1*/*HHF2* cluster multialignments in Figure S4. Because these sequences are duplicated in opposite chromosomal orientations, multiple forward and reverse reads are observed and aligned on each of the homologous regions on chromosomes II and XIV, as expected. Formally, the presence of both forward and reverse reads at a given position places this region within the M category. Forward/reverse strand multialignments come from various sources. They can reflect stochastic sequence redundancies between chromosomes or local head to tail repeats. In yeast, this includes the highly repetitive Ty elements that integrate within a chromosome, irrespective of strand orientation. The paucity of inverted repeats likely reflects their spontaneous instability that generates translocation events (Lemoine *et al.* 2005) when DNA replication is perturbed. Also, a few duplicated regions, reminiscent of the ancestral duplication of the *S. cerevisiae* whole-genome (Wolfe and Shields 1997; Kellis *et al.* 2004) and its evolutionarily selected and drifted remnants contain constitutive M regions. Altogether, using 50 nt-reads and allowing up to six mismatches, we could estimate that the cumulative sum of the M regions corresponds to 8.4% of the genome (approximately 10^6^ nt), comprising 1823 M regions of various lengths (50−28,934 nt), dispersed within all of the chromosomes. Consistently, the sum of the M regions, defined from our experimental HTS data was 8.7%. This slight difference is likely explained by the production of experimental reads containing sequencing errors that are sufficient to generate higher noise and further multialigned reads.

### SNVs annotation

Multialigned reads are either fully identical to the reference sequence, or slightly polymorphic because of nearly exact repetitive sequences. We then asked whether we could use these variations, since they provide specific sequence signatures that can be used to mark some, if not all, members of a given repeat family. To do so, we identified and annotated all of the base changes from multialigned simulated reads obtained from virtual HTS mapping (Figure 1D) and then used these base change annotations as predictive molecular markers to deconvolute the M reads. This annotation, available at SGD, provides a new resource to analyze the diversity of the yeast M subgenome and precisely pinpoints variable positions within repetitive sequences.

### G-deNoise filtering

**Figure 5:**
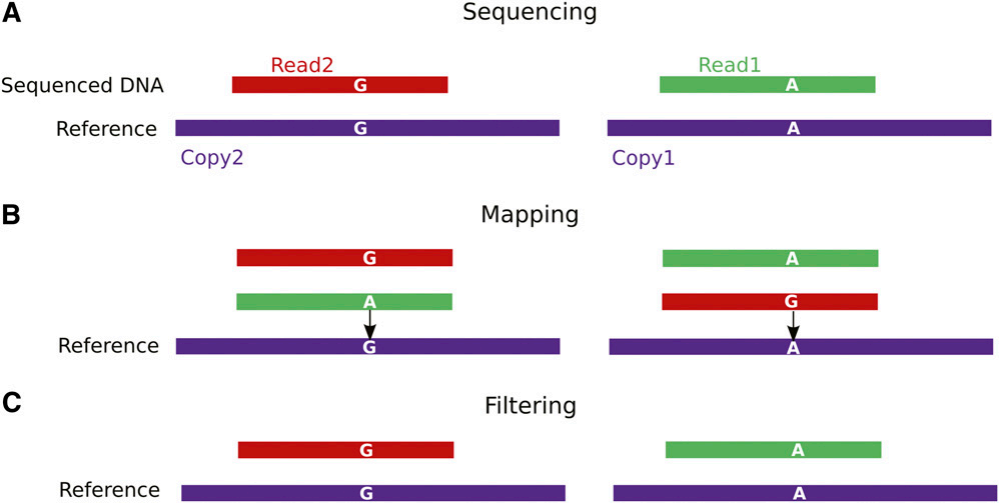
Principle of g-deNoise filtering. Illustration with two near-exact copies of the same repetitive sequence. (A) Read1 and Read2 are sequenced on two copies of the same repetitive sequence. Copy1 and Copy2 are intragenomic repeats that differ by a single nucleotide (A↔G). (B) After mapping, and before any filtering, Read1 and Read2 are multialigned. Each of these reads has one perfect alignment at the genomic position from which it originates, and one mismatched alignment at the position of the polymorphic copy. The mismatched position corresponds to an intragenomic SNV, preannotated in multialignments of virtual reads. (C) Filtering by g-deNoise consists of discarding alignments allowing such intra SNVs and therefore avoids the miscalling of an acquired mutation. SNV, single-nucleotide variant.

The aforementioned SNVs annotation led us to set up a new bioinformatics method, that we termed g-deNoise, to efficiently filter polymorphic multialigned reads generated from virtual and experimental sequencing data. As illustrated in Figure 5, reads generated from a duplicated region containing a single base change (Figure 5A) align to both regions (Figure 5B). The principle of g-deNoise, is to use the preannotated SNV as a specific signature to distinguish the source of the individual reads (Figure 5C). G-deNoise differs from a “Best match” strategy because its filtering principle is only based on the presence of a predicted SNV within the alignments, rather than a best match that is sensitive to the potential presence of an unpredicted sequence variation. First, performing g-deNoise on the virtual HTS mapping allowed us to identify certain regions (M_U_) containing specific polymorphisms that became uniquely aligned, after filtering. These could then be removed and the regions that remained multi-aligned were named M_M_. For example, Figure S4 illustrates our annotation of the duplicated *HHF1/HHF2* genes. As expected, in the virtual HTS mapping, the reads from the duplicated *HHF2* gene align with the reverse strand and exhibit the expected eight SNVs that uniquely distinguish these duplicated genes (Figure S4B). Furthermore, our experimental reads from the line wt_A100, containing these eight SNVs, were also clearly detected (Figure S4C). In summary, the effect of the g-deNoise filter on the initial *HHF1* multialignments identifies the internal M_U_ regions (Figure S4E), thus becoming prone to a robust detection of potential variants.

**Figure 6:**
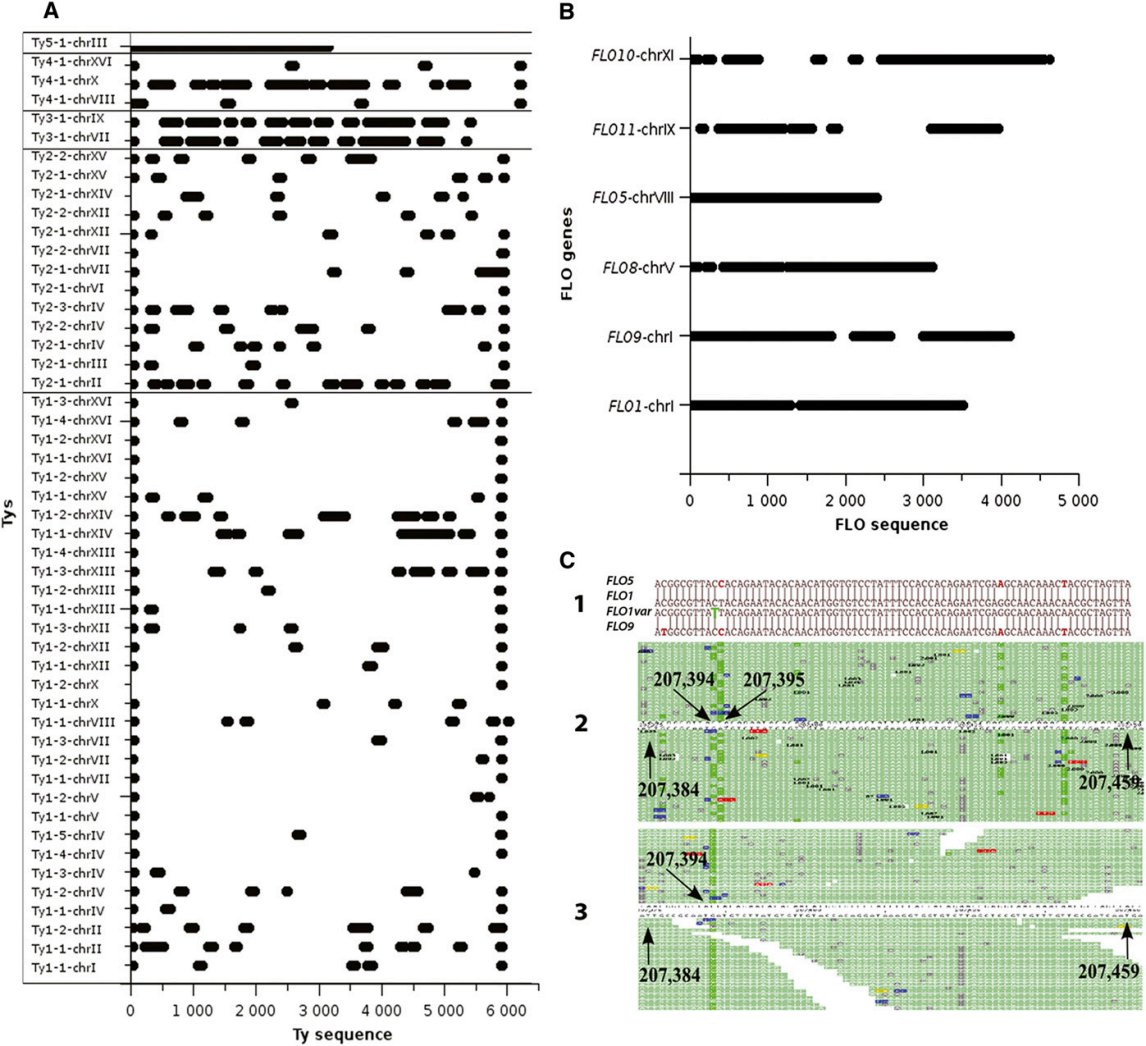
Analysis of M regions from two classes of repetitive elements. (A) M_U_ regions overlapping the Ty1-Ty5 elements. (B) M_U_ regions overlapping the six *FLO* gene copies. The M_U_ regions are represented by horizontal bold bars. (C) Detection of acquired mutations within the polymorphic *FLO1*, *FLO5*, and *FLO9* multi-copy genes. (1) Alignment of the *FLO5*, *FLO1*, *FLO9*, and *FLO1* variant (identified here) gene sequences. (2) Visualization of the forward and reverse strand read mapping of the *FLO1* gene on the chromosome I in the experimental HTS from the wt_A100 wild type line with the SOLiD Alignment Browser. The coordinates of the regions are indicated (arrows). The fluorescent green bars outline positions of mismatches consistent with a base change. (3) Same as (2) after using g-deNoise. The reads containing the intragenomic SNVs were removed and the unpredicted (acquired) SNP at position 207,394 persisted. The nature and position of the acquired mutation is indicated in green in the *FLO1* variant sequence in (1). This identification of this acquired SNP was based on the examination of the 50 nt-reads, containing the adjacent *FLO1* specific polymorphisms. It was also confirmed by Sanger sequencing of a polymerase chain reaction product amplified using primers designed in the U regions flanking the *FLO1* gene. SNP, single-nucleotide polymorphism.

As a second example, we chose to identify sequence variations in the highly polymorphic retrotransposon regions. The reference genome contains 50 copies of these Ty1, Ty2, Ty3, Ty4, or Ty5 6-kb elements. The results illustrated in Figure 6 and Figure S5 show that although several M_M_ regions of variable length remain indistinguishable between Ty copies, numerous M_U_ regions exist.

Analyzing 50 nt and six mismatches per read genome-wide, we estimated that 614,530 bp (40% of the total length of the M regions) of the *S. cerevisiae* genome contain M_U_ regions. Overall, we now estimate that the robust analysis of the U+M_U_ regions reach 94.96% of the *S. cerevisiae* genome.

### Application to the detection of SNP variants in the U and M regions

Sequencing of four wild-type *S. cerevisiae* mutation accumulation cell lines of the BY4741 strain background, led us to examine the presence of SNPs both within the U and M regions. Using the Bioscope pipeline and the 2008 S288c reference sequence, we first examined the U regions. We identified 408 SNPs that were present in all four lines (constitutive). Consistently, 357 SNPs (87.5%) were corrected in the 2011 S288c reference genome (current release). Soon after, partial sequences from the BY4741 and BY4742 yeast strains, that comprise 14 of the 16 total chromosomes, have been provided on SGD (BY4741_Toronto_2012.fsa and BY4742_Toronto_2012.fsa). Among the 51 SNPs that we identified to differ between BY4741 and S288c, 38 were also present in the BY genome releases. We confirmed that they represent true polymorphisms that distinguish the BY and S288c strain backgrounds. To further assess the remaining 13 SNPs, we performed Sanger sequencing and in each case confirmed our NGS call.

Concerning the acquired variants (present in only one out of the four lines) we identified 39 SNPs in the U regions. In the M regions, we used g-deNoise to filter multi-alignments. We identified two constitutive SNPs in the *YBLWTy2-1* element on chromosome II (C > T, position 30,012 and A > G, position 30,050) and two acquired SNPs, present in only one of the four wild-type lines. Precisely, we identified a C > T at position 207,394 on chromosome I in the *FLO1* gene (Figure 6C) and a G > C at position 156,571 on chromosome X in the *RPS21B* gene encoding the small (40S) ribosomal subunit, in the lines wt_A100 and the wt_B100, respectively. These four SNPs were confirmed by Sanger sequencing of a PCR-product amplified using primers of the U regions flanking the SNPs. Altogether, these results demonstrate the power of using the preannotated HTS profile of the U and M regions to robustly identify sequence variations. The lists of the constitutive and acquired SNPs identified in the present studies of the commonly used BY4741 strain are reported in Table S2 and Table S3.

## Discussion

Herein, we report novel methods for SNPs calling within unique and repeated genomic regions. These methods rely on two complementary bioinformatics strategies: the production of simulated HTS experiments to generate virtual HTS profiles, allowing us to define U and M regions of the genome, whereas in a second step, the “g-deNoise” filtering is used to discriminate the M_M_ and M_U_ regions. Concerning the analyses of the experimental data, our alignment filtering consists of (i) discarding alignments consistent with a SNV from intragenomic origins, (ii) retaining the unique alignments, and (iii) safely analyzing the U and M_U_ regions. Compared with other filters using “best alignment” strategies as SOAP (Li *et al.* 2008b) and PASS (Campagna *et al.* 2009) mapping programs that filter experimental data, the advantage of our method is to use noise-less simulated reads for the preannotation of the intragenomic SNVs. Compared with GMS (Lee and Schatz 2012), that associates a probability to each position of the genome that pinpoints noisy repetitive regions but does not propose an ultimate FP binary status such as “is” or “is not” to each call, our approach is decisive and thereof facilitates the detection of alternative variations at the same position (for example, if there is an A in the reference sequence but a T in a repeated region, we are able to identify C or G changes if an acquired SNP is present in an experimental sample). Other applications could be derived from the establishment of virtual HTS profiles; for example, the filtering of small insertions/deletions and rearrangements, as well as for the identification of copy number changes when combined with the analysis of experimental HTS coverage. It can be implemented for whole genome, exome, and draft sequences. The knowledge of the intragenomic SNVs can also optimize the filtering of low-coverage cases. To follow the specification of the experimental reads in terms of length and quality that are in constant evolution, the parameters of virtual HTS profile determinations can be modulated.

Concerning the analyses of the repetitive regions, the ability to identify the M_U_ regions and use their internal polymorphisms, provides a significant progress, but due to the lack of polymorphisms, the M_M_ regions remain difficult to analyze. In these regions, the occurrence of acquired polymorphisms can be identified, but their ability to be mapped to a given unique repeat remains a challenge. Our pair-ended libraries that generated two reads separated by ~200 bp, were insufficient to resolve certain multialignments because many repetitive sequences are greater than 200 bp. The usage of mate-paired libraries with larger inserts between the reads or extra-long single molecule read technologies (Sharon *et al.* 2013) associated with powerful *de novo* assembly software will be necessary to be exhaustive.

Our analysis of the *S. cerevisiae* genome is a resource of general interest available at SGD. This resource will have multiple benefits: (i) to define the U and M regions, (ii) to provide a simple and global U/M readout of the genome complexity that can be used to compare variant strains and species, (iii) to allow precise measurements of HTS coverage by using the set of U regions as the reference genome ploidy index to count experimental reads, and (iv) to facilitate the identification of constitutive and acquired mutations in both unique and highly repetitive regions of variant strains.

## Supplementary Material

Supporting Information
