# Supplementary material for: Sequence Profiling of the Saccharomyces cerevisiae Genome Permits Deconvolution of Unique and Multialigned Reads for Variant Detection
Source: G3 (Bethesda). 2014 Feb 20;4(4):707–15. doi: 10.1534/g3.113.009464 (PMC4059241; doi:10.1534/g3.113.009464)
Supplement: Supporting Information [file supp_4_4_707__index.html]

Sequence Profiling of the Saccharomyces cerevisiae Genome Permits Deconvolution of Unique and Multialigned Reads for Variant Detection — Supporting Information 

# Sequence Profiling of the *Saccharomyces cerevisiae* Genome Permits Deconvolution of Unique and Multialigned Reads for Variant Detection

## Supporting Information for Jubin *et al.*, 2014

**Files in this Data Supplement:**

- Supporting Information - Figures S1-S5, File S1, and Tables S1-S3 (PDF, 517 KB)
- Figure S1 - M regions visualization through SGD browser (http://www.yeastgenome.org/). (PDF, 192 KB)
- Figure S2 - Virtual HTS profile coverage of chromosome II. (PDF, 567 KB)
- Figure S3 - Forward (F) and reverse (R) strand coverage of 50 nt-reads virtual HTS profile along the whole chromosome II. (PDF, 158 KB)
- Figure S4 - Application of the g-deNoise filtering on the duplicated histone H4 *HHF1* multi-aligned regions. (PDF, 386 KB)
- Figure S5 - Visualization of reads mapping on the M regions of *YBLWTy2-1*. (PDF, 152 KB)
- Table S1 - Functional annotation of M regions along chromosome II. (PDF, 120 KB)
- Table S2 - Constitutive SNPs found in the U regions in the wild-type mutation accumulation lines. (PDF, 120 KB)
- Table S3 - Acquired SNPs found in the U regions in the wild-type mutation accumulation lines. (PDF, 121 KB)
- File S1 - Supporting data (.zip, 852 KB)
