# Supplementary material for: Sequence Profiling of the Saccharomyces cerevisiae Genome Permits Deconvolution of Unique and Multialigned Reads for Variant Detection
Source: G3 (Bethesda). 2014 Feb 20;4(4):707–15. doi: 10.1534/g3.113.009464 (PMC4059241; doi:10.1534/g3.113.009464)
Supplement: Supporting Information [file supp_g3.113.009464_009464SI.pdf]

G3 · Genes | Genomes | Genetics

Sequence profiling of the *Saccharomyces cerevisiae* genome permits deconvolution of unique and multi-aligned reads for variant detection

Claire Jubin[*], Alexandre Serero[*], Sophie Loeillet[*], Emmanuel Barillot[§] and Alain Nicolas[*,1]

[*]Institut Curie Centre de Recherche, UMR3244 CNRS, Université Pierre et Marie Curie, 75248 Paris Cedex 05, France,[§]Institut Curie Centre de Recherche, INSERM U900, 75248 Paris, France. Ecole des Mines Paris Tech, 77300 Fontainebleau, France.

[1]Corresponding author: Alain Nicolas. Institut Curie Centre de Recherche, UMR3244 CNRS, 26 rue d'Ulm 75248 Paris Cedex 05, France. +33 1 56 24 65 20. E-mail: alain.nicolas@curie.fr.

Data are available at http://www.ncbi.nlm.nih.gov/Traces/sra with accession number: SRR1014750, SRR1014753, SRR1014752 and SRR1014754.

DOI: 10.1534/g3.113.009464

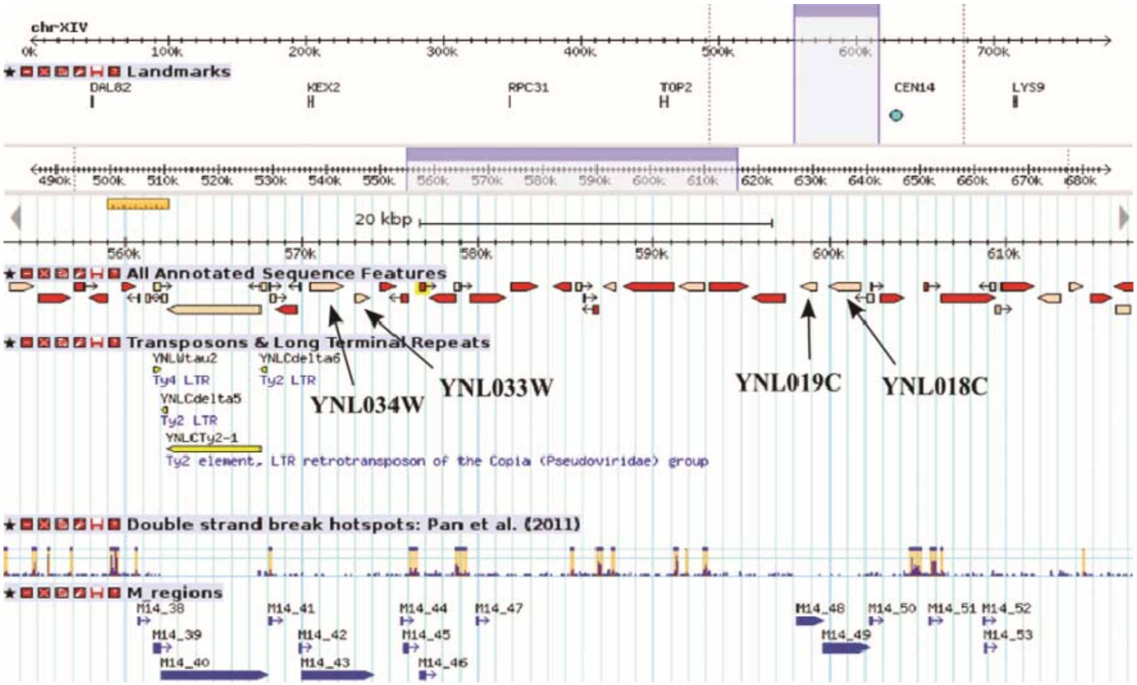

Figure S1 M regions visualization through SGD browser (http://www.yeastgenome.org/). M regions are represented as blue arrows on the last track. M regions may correspond to already known repeated elements such as Ty and LTR elements (M14_40) or to multi-copy genes. For example, the *YNL034W* (570,477-572,315) and *YNL033W* (572,999-573,853) loci, covered by M14_43, are duplications of the locus *YNL018C* (601,774-599,936) covered by M14_49, and the locus *YNL019C* (599,230-598,376) covered by M14_48, respectively. The SGD interface includes functional annotations; for example, the meiotic “Double strand break hotspots: Pan et al. (2011)”.

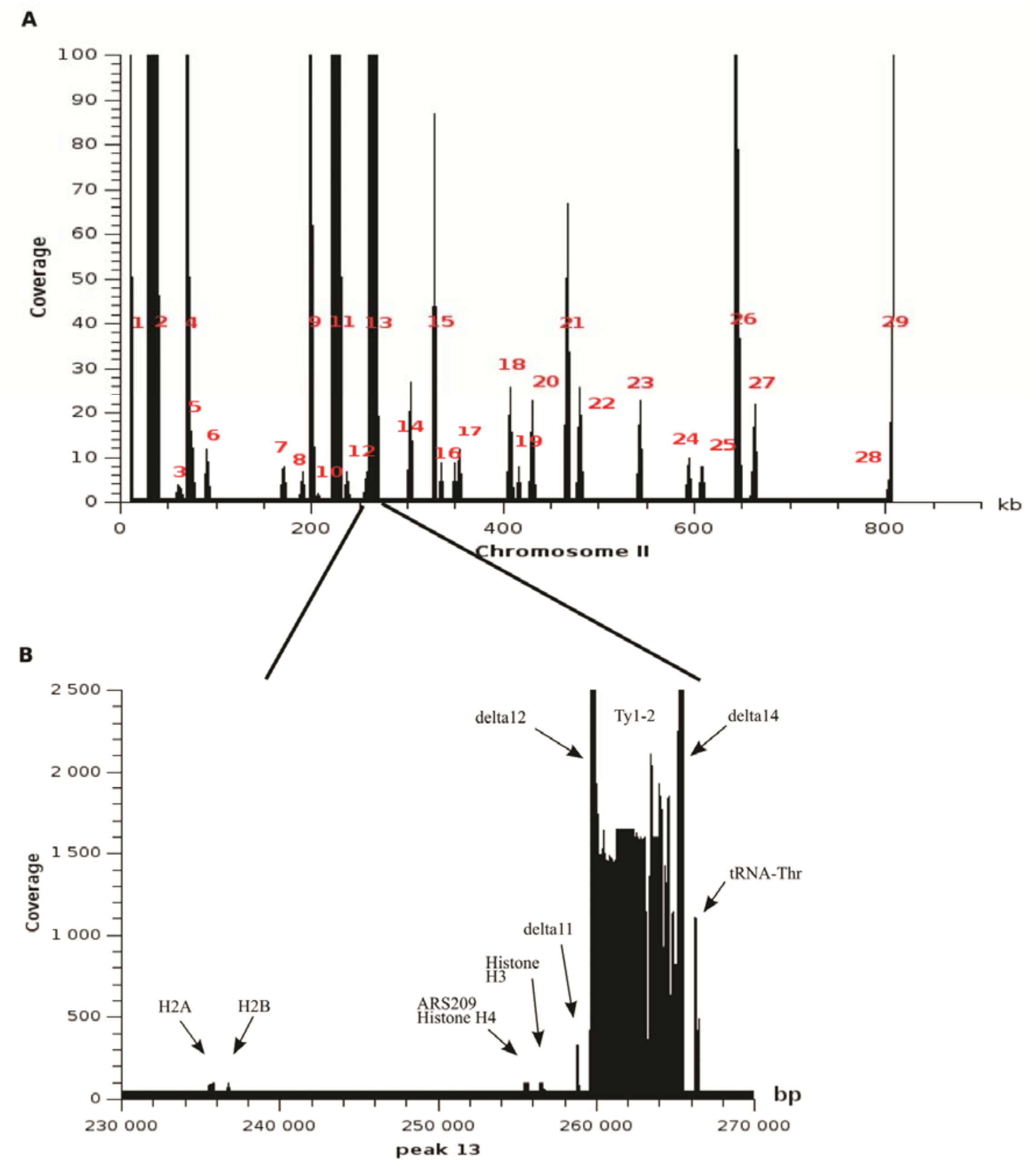

Figure S2 Virtual HTS profile coverage of chromosome II. (A) 2kb window coverage along the whole chromosome II, computed at the base level. (B) Contribution of functional annotation to multi-aligned regions of the peak 13.

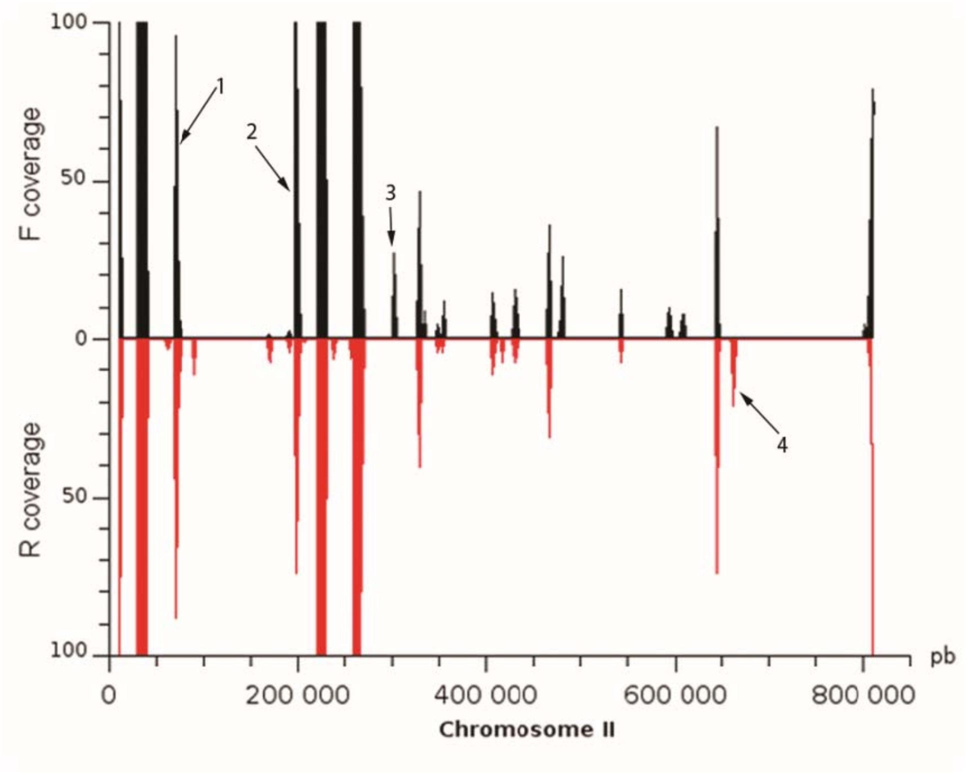

Figure S3 Forward (F) and reverse (R) strand coverage of 50 nt-reads virtual HTS profile along the whole chromosome II. Some regions are repetitive on the both strands (1, 2) and some others are only repetitive on one strand (3, 4). This figure illustrates that virtual HTS profile is able to discriminate repetitiveness according to the strand in the reference genome.

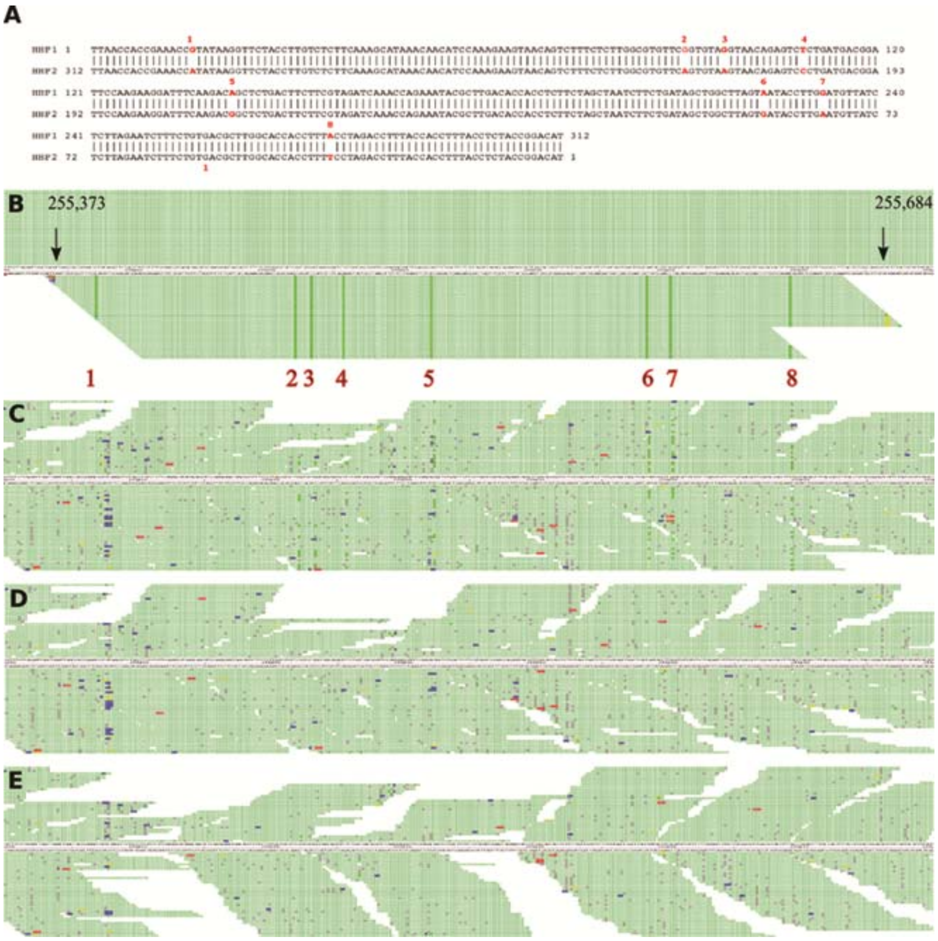

Figure S4 Application of the g-deNoise filtering on the duplicated histone H4 *HHF1* multi-aligned regions. (A) Pairwise alignments of *HHF1* (chr. II: 255,373-255,684) and *HHF2* (chr. XIV: 576,727-577,038). The two genes, *HHF1* and *HHF2,* exhibit eight base changes (numbered). (B) Multi-aligned simulated reads. As *HHF1* and *HHF2* are inverted copies, reads generated on *HHF1* are aligned on the forward strand and reads generated on *HHF2* are aligned on the reverse strand. (C) Multi-aligned reads in experimental data. Light green, yellow and red positions suggest one, two and three base changes respectively. Grey and blue positions: sequencing errors. (D) Alignments of experimental data after discarding alignments consistent with an intra SNV, and (E) remaining unique-alignments after discarding multi-alignments in (D). Here, with 50 nt-reads, we observe that the whole gene sequence is still covered by alignments and is therefore now reduced into a M_u region prone to robust SNP polymorphism detection.

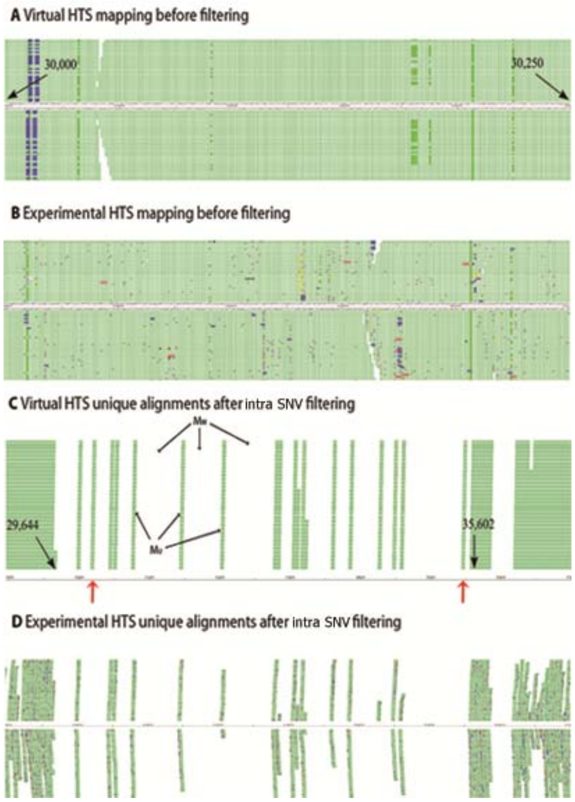

Figure S5 Visualization of reads mapping on the M regions of *YBLWTy2-1*. Alignments without filtering are represented along the 30,000-30,250 sequence of *YBLWTy2-1* (chr. II: 29,644-35,602) containing the TYA Gag gene, obtained in (A) the virtual HTS profile and in (B) the experimental HTS data. The (C) and (D) panels represent the M_U regions along the *YBLWTy2-1* (~6kb) sequence in the virtual HTS profile and corresponding HTS experimental data, respectively. Note that two M_U regions peaks, indicated by red arrows in virtual HTS profile (C), are missing in the HTS experimental data (D). This indicates the loss of specific polymorphism in the experimental sample, probably a loss or gain of an intra SNVs.

File S1

Supporting data

File S1 is available for download as a .zip file at http://www.g3journal.org/lookup/suppl/doi:10.1534/g3.113.009464/-/DC1

Table S1 Functional annotation of M regions along chromosome II

#peak	M region start	M region stop	Systematic name	Standard name
**1**	1	5856	TEL02L	
**1**			TEL02L-YP	
**1**			YBL113C	
**1**			YBL113W-A	
**1**			TEL02L-YP_repeat_region	
**1**			YBL112C	
**1**			YBL111C	
**1**			YBL109W	
**1**			TEL02L-XR	
**1**	5905	8310	YBL109W	
**1**			TEL02L-XR	
**1**			TEL02L-XC	
**1**			YBL108C-A	*PAU9*
**1**			YBL108W	
**1**	8328	8420	YBL108W	
**1**	8864	8915	YBLWdelta2	
**1**	8944	9012	YBLWdelta2	
**1**	9080	9434	YBLWdelta3	
**1**	9569	9684	tL(UAA)B1	
**2**	29623	35611	YBLWdelta4	
**2**			YBLWty2-1	
**2**			YBLWdelta5	
**2**			YBWdelta6	
**2**	35838	36229	YBLCtau1	
**2**	36385	36496	tF(GAA)B	
**3**	59834	60099	YBL087C	*RPL23A*
**3**	60101	60192	YBL087C	*RPL23A*
**4**	68267	68356	YBL084C	*CDC27*
**5**	72337	72410	YBL081C	
**6**	88512	89126	YBL072C	*RPS8A*
**6**	92826	92875	YBL068W	*PRS4*
**7**	166365	166436	YBL029W	
**7**	168808	168889	YBL027W	*RPL19B*
**7**	168927	169201	YBL027W	*RPL19B*
**7**	169209	169379	YBL027W	*RPL19B*
**7**	175875	175929	YBL023C	*MCM2*
**8**	187312	187368	YBL017C	*PEP1*
**8**	187673	187781	YBL017C	*PEP1*
**8**	187991	188054	YBL017C	*PEP1*
**8**	188351	188418	YBL017C	*PEP1*
**8**	188477	188590	YBL017C	*PEP1*
**8**	188592	188742	YBL017C	*PEP1*
**8**	188877	188928	YBL017C	*PEP1*
**8**	188930	188994	YBL017C	*PEP1*
**8**	189002	189051	YBL017C	*PEP1*
**9**	197488	197583	tI(AAU)B	
**10**	197616	198069	tG(GCC)B	
**10**			YBLWsigma1	
**11**	205746	205813	YBL011W	*SCT1*
**12**	221028	226963	YBLWdelta8	
**12**			YBLWdelta9	
**12**			YBLWTy1-1	
**12**			YBLWdelta10	
**12**	227062	227177	tS(AGA)B	

**13**	235403	235810	YBL003C	*HTA2*
**13**	236618	236763	YBL002W	*HTB2*
**13**	255367	255688	ARS209	*ARS209*
**13**			YBR009C	*HHF1*
**13**	256374	256624	YBR010W	*HHT1*
**13**	256683	256737	YBR010W	*HHT1*
**13**	258689	258810	YBRCdelta11	
**13**	258859	258926	YBRCdelta11	
**13**	259564	263149	YBR012C	
**13**			YBRWdelta12	
**13**			YBRWTy1-2	
**13**	263164	265504	YBRWTy1-2	
**13**	266172	266256	YBRCdelta14	
**13**	266367	266465	tT(AGU)B	
**13**	270424	270488	YBR016W	
**14**	300159	301247	YBR031W	*RPL4A*
**15**	326780	326872	tV(UAC)B	
**15**	327163	327352	YBRWdelta15	
**16**	332826	332880	YBR048W	*RPS11B*
**16**	333383	333810	YBR048W	*RPS11B*
**16**	343123	343181	YBR054W	*YRO2*
**16**	343564	343616	YBR054W	*YRO2*
**17**	347591	347704	/	
**17**	350815	350906	tQ(UUG)B	
**17**	353582	353664	/	
**17**	372325	372392	YBR067C	*TIP1*
**18**	405845	406059	tR(UCU)B	
**18**			tD(GUC)B	
**19**	414180	414350	YBR084C-A	*RPL19A*
**19**	414358	414632	YBR084C-A	*RPL19A*
**19**	414670	414751	YBR084C-A	*RPL19A*
**20**	427755	428109	YBR092C	*PHO3*
**20**	428336	428399	YBR092C	*PHO3*
**20**	428485	428576	YBR092C	*PHO3*
**20**	428671	428830	YBR092C	*PHO3*
**20**	428883	428984	YBR092C	*PHO3*
**20**	429576	429959	YBR093C	*PHO5*
**20**	430186	430249	YBR093C	*PHO5*
**20**	430335	430426	YBR093C	*PHO5*
**20**	430521	430680	YBR093C	*PHO5*
**20**	430698	430834	YBR093C	*PHO5*
**20**	430881	430936	YBR093C	*PHO5*
**21**	463997	464290	YBR112C	*CYC8*
**22**	477659	479049	YBR118W	*TEF2*
**23**	541431	541526	YBR150C	*TBS1*
**23**	541577	541639	YBR150C	*TBS1*
**24**	591706	592422	YBR181C	*RPS6B*
**25**	604937	605061	YBR189W	*RPS9B*
**25**	605069	605152	YBR189W	*RPS9B*
**25**	605195	605451	YBR189W	*RPS9B*
**25**	606653	606731	YBR191W	*RPL21A*
**25**	606734	606918	YBR191W	*RPL21A*
**25**	606920	607131	YBR191W	*RPL21A*
**25**	627307	627364	YBR202W	*MCM7*
**26**	642988	643084	tC(GCA)B	
**26**	643470	643865	YBRCtau2	
**26**			YBRWdelta18	
**26**	645147	645250	tE(UUC)B	
**26**	646075	646133	/	

**27**	659261	659328	YBR218C	*PYC2*
**27**	659576	659630	YBR218C	*PYC2*
**27**	659645	659730	YBR218C	*PYC2*
**27**	659774	659853	YBR218C	*PYC2*
**27**	660212	660594	YBR218C	*PYC2*
**27**	660610	660759	YBR218C	*PYC2*
**27**	660791	660948	YBR218C	*PYC2*
**27**	661274	661362	YBR218C	*PYC2*
**27**	661406	661455	YBR218C	*PYC2*
**27**	661460	661757	YBR218C	*PYC2*
**27**	661835	661965	YBR218C	*PYC2*
**27**	701990	702056	/	
**27**	780350	780468	YBR289W	*SNF5*
**28**	801690	801919	YBR297W	*MAL33*
**29**	804863	808394	YBR298C-A	
**29**			YBR299W	*MAL32*
**29**	808396	813178	YBR300C	
**29**			YBR301W	*PAU24*
**29**			YBR302C	*COS2*

Table S2 Constitutive SNPs found in the U regions in the wild-type mutation accumulation lines

Line	Chrom.	Position	ORF	Gene	Ref.	Variant	Aa change	Sequence Ontology term
wt_ABCE100	chrII	15 101	YBL105C	*PKC1*	T	G	I866L	missense_variant
wt_ABCE100	chrIII	143 131	Intergenic	-	T	C	-	intergenic_variant
wt_ABCE100	chrIII	162 361	Intergenic	-	T	C	-	intergenic_variant
wt_ABCE100	chrIII	162 640	YCR024C-B	YCR024C-B	T	G	S76R	missense_variant
wt_ABCE100	chrIII	162 694	YCR024C-B	YCR024C-B	G	A	synonymous	synonymous_variant
wt_ABCE100	chrIII	163 059	YCR024C-A	*PMP1*	T	C	synonymous	synonymous_variant
wt_ABCE100	chrV	48 384	Intergenic	-	T	C	-	intergenic_variant
wt_ABCE100	chrV	154 531	YER001W	*MNN1*	T	A	S338T	missense_variant
wt_ABCE100	chrV	352 394	YER096W	*SHC1*	A	G	K233E	missense_variant
wt_ABCE100	chrV	517 529	Intergenic	-	T	C	-	intergenic_variant
wt_ABCE100	chrXIII	448 333	Intergenic	-	G	A	-	intergenic_variant
wt_ABCE100	chrXIII	680 936	YMR207C	*HFA1*	T	C	K877E	missense_variant
wt_ABCE100	chrXIII	680 940	YMR207C	*HFA1*	C	T	synonymous	synonymous_variant

Table S3 Acquired SNPs found in the U regions in the wild-type mutation accumulation lines

Line	Chrom.	Position	ORF	Gene	Ref.	Variant	Aa change	Sequence Ontology term
wt_A100	chrII	468 275	YBR114W	*RAD16*	C	T	T343M	missense_variant
wt_B100	chrIII	1 989	YCL076W	YCL076W	G	A	G200S	missense_variant
wt_B100	chrIV	191 155	YDL147W	*RPN5*	C	A	Q78K	missense_variant
wt_A100	chrIV	573 098	YDR060W	*MAK21*	A	T	D817V	missense_variant
wt_B100	chrIV	738 528	YDR141C	*DOP1*	A	C	I490M	missense_variant
wt_A100	chrIV	866 198	YDR207C	*UME6*	C	T	R442Q	missense_variant
wt_E100	chrIV	1 171 524	YDR348C	*PAL1*	G	A	P101L	missense_variant
wt_E100	chrIV	1 452 784	YDR501W	*PLM2*	G	T	E478STOP	stop_gained
wt_B100	chrIV	1 486 605	YDR523C	*SPS1*	G	T	A145D	missense_variant
wt_C100	chrV	234 495	YER041W	*YEN1*	T	C	S679P	missense_variant
wt_C100	chrV	312 202	Intergenic	-	A	G	-	intergenic_variant
wt_E100	chrV	404 934	YER123W	*YCK3*	A	G	Q41R	missense_variant
wt_C100	chrVII	313 763	YGL100W	*SEH1*	G	T	R177I	missense_variant
wt_E100	chrVII	376 132	YGL067W	*NPY1*	A	T	H11L	missense_variant
wt_C100	chrVII	1 041 377	YGR274C	*TAF1*	G	T	synonymous	synonymous_variant
wt_B100	chrVIII	137 015	YHR016C	*YSC84*	G	A	P425S	missense_variant
wt_A100	chrVIII	275 555	YHR084W	*STE12*	A	G	Y461C	missense_variant
wt_A100	chrVIII	368 794	YHR132C	*ECM14*	A	G	L334S	missense_variant
wt_A100	chrVIII	390 799	YHR146W	*CRP1*	C	T	T167I	missense_variant
wt_A100	chrX	618 251	YJR101W	*RSM26*	G	A	synonymous	synonymous_variant
wt_A100	chrXI	8 145	Intergenic	-	G	C	-	intergenic_variant
wt_A100	chrXI	291 061	Intergenic	-	C	T	-	intergenic_variant
wt_B100	chrXI	480 315	YKR021W	*ALY1*	A	C	E361A	missense_variant
wt_E100	chrXII	197 723	YLR027C	*AAT2*	T	A	K121N	missense_variant
wt_B100	chrXII	318 957	YLR089C	*ALT1*	C	A	Q353H	missense_variant
wt_C100	chrXII	357 349	YLR106C	*MDN1*	G	A	synonymous	synonymous_variant
wt_E100	chrXII	360 374	YLR106C	*MDN1*	T	G	Y1122S	missense_variant
wt_B100	chrXII	837 516	Intergenic	-	C	T	-	intergenic_variant
wt_E100	chrXII	935 171	YLR409C	*UTP21*	G	C	S688W	missense_variant
wt_E100	chrXII	1 034 119	YLR450W	*HMG2*	T	G	F498C	missense_variant
wt_B100	chrXIII	470 741	YMR102C	YMR102C	T	A	S538C	missense_variant
wt_A100	chrXIII	912 702	Intergenic	-	G	T	-	intergenic_variant
wt_B100	chrXIV	275 036	YNL193W	YNL193W	G	A	synonymous	synonymous_variant
wt_C100	chrXIV	752 345	YNR065C	YNR065C	G	C	S452R	missense_variant
wt_A100	chrXV	406 610	Intergenic	-	T	C	-	intergenic_variant
wt_C100	chrXV	594 444	Intergenic	-	A	T	-	intergenic_variant
wt_C100	chrXVI	120 124	Intergenic	-	A	T	-	intergenic_variant
wt_A100	chrXVI	307 821	YPL128C	*TBF1*	G	A	T134I	missense_variant
wt_A100	chrXVI	510 123	Intergenic	-	G	T	-	intergenic_variant
