# Supplementary material for: Sequence Profiling of the Saccharomyces cerevisiae Genome Permits Deconvolution of Unique and Multialigned Reads for Variant Detection
Source: G3 (Bethesda). 2014 Feb 20;4(4):707–15. doi: 10.1534/g3.113.009464 (PMC4059241; doi:10.1534/g3.113.009464)
Supplement: Supporting Information [file supp_g3.113.009464_FigureS1.pdf]

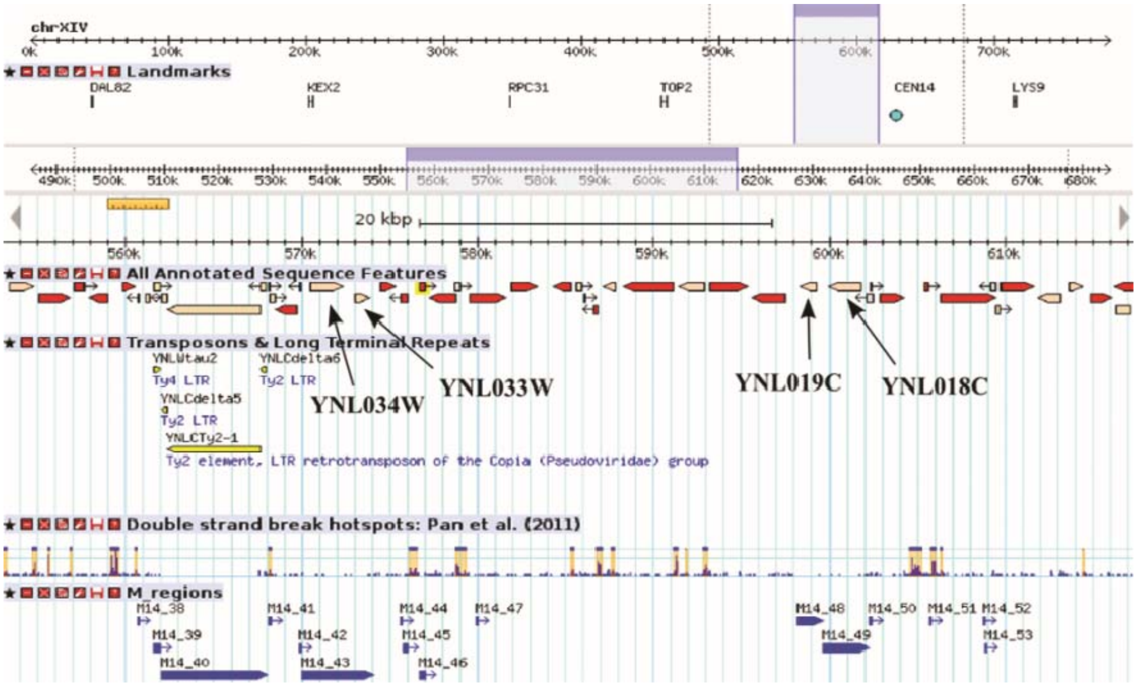

Figure S1 M regions visualization through SGD browser (http://www.yeastgenome.org/). M regions are represented as blue arrows on the last track. M regions may correspond to already known repeated elements such as Ty and LTR elements (M14_40) or to multi-copy genes. For example, the *YNL034W* (570,477-572,315) and *YNL033W* (572,999-573,853) loci, covered by M14_43, are duplications of the locus *YNL018C* (601,774-599,936) covered by M14_49, and the locus *YNL019C* (599,230-598,376) covered by M14_48, respectively. The SGD interface includes functional annotations; for example, the meiotic “Double strand break hotspots: Pan et al. (2011)”.
