# Supplementary material for: Sequence Profiling of the Saccharomyces cerevisiae Genome Permits Deconvolution of Unique and Multialigned Reads for Variant Detection
Source: G3 (Bethesda). 2014 Feb 20;4(4):707–15. doi: 10.1534/g3.113.009464 (PMC4059241; doi:10.1534/g3.113.009464)
Supplement: Supporting Information [file supp_g3.113.009464_FigureS2.pdf]

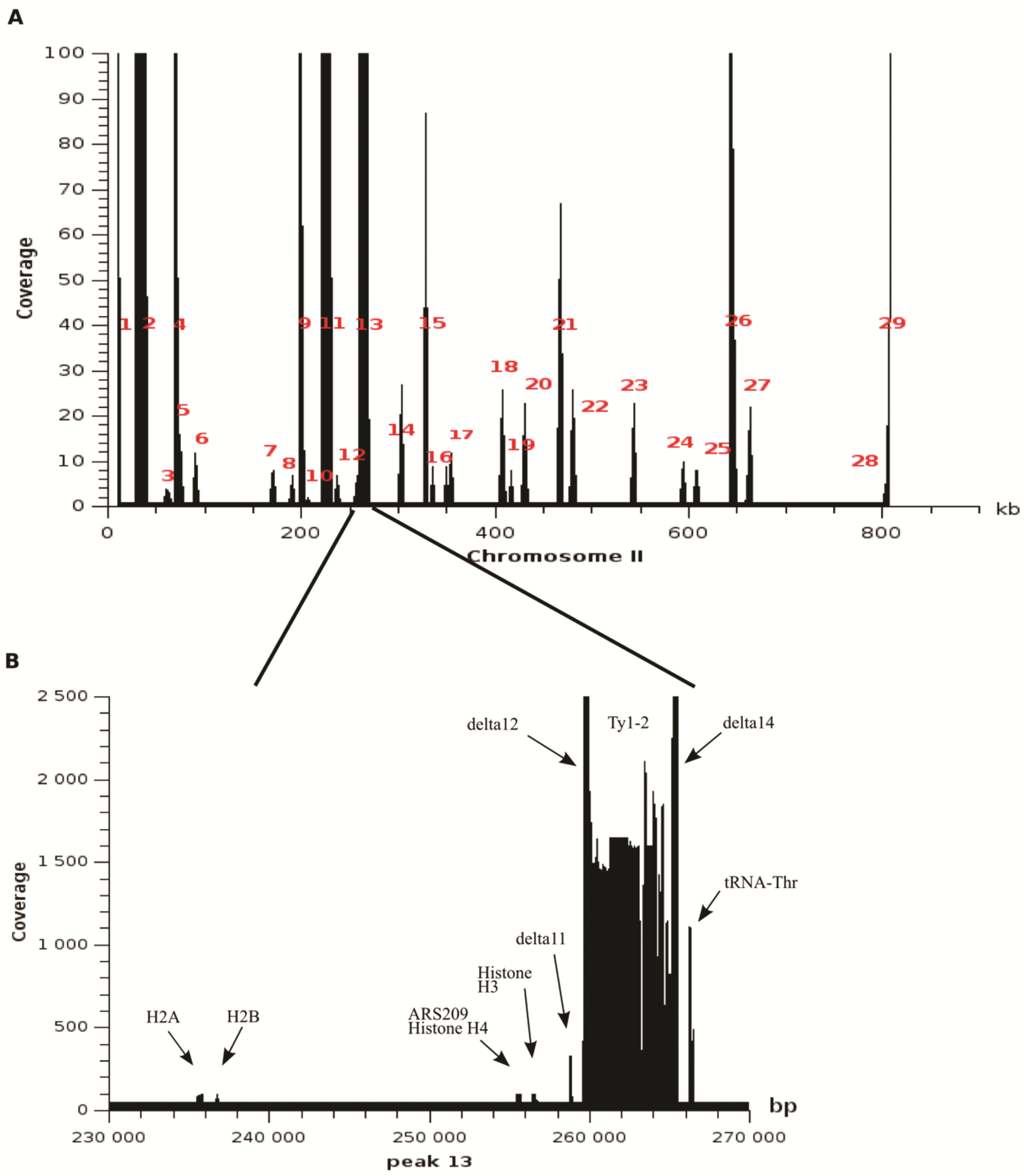

Figure S2 Virtual HTS profile coverage of chromosome II. (A) 2kb window coverage along the whole chromosome II, computed at the base level. (B) Contribution of functional annotation to multi-aligned regions of the peak 13.
