# Supplementary material for: Sequence Profiling of the Saccharomyces cerevisiae Genome Permits Deconvolution of Unique and Multialigned Reads for Variant Detection
Source: G3 (Bethesda). 2014 Feb 20;4(4):707–15. doi: 10.1534/g3.113.009464 (PMC4059241; doi:10.1534/g3.113.009464)
Supplement: Supporting Information [file supp_g3.113.009464_FigureS3.pdf]

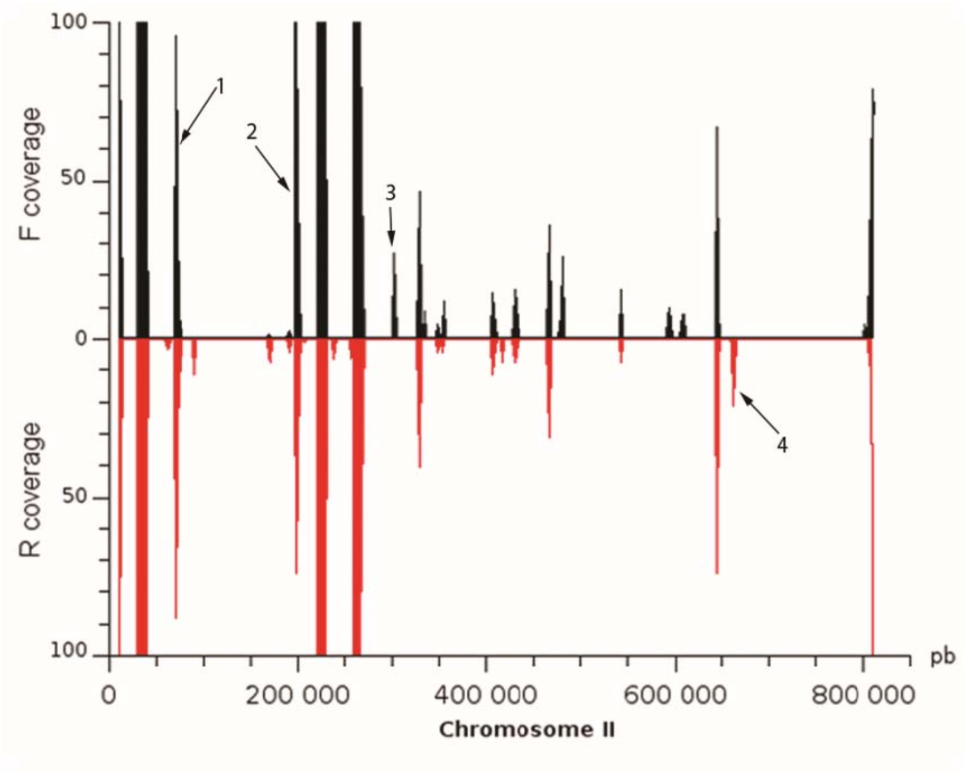


**Figure S3** Forward (F) and reverse (R) strand coverage of 50 nt-reads virtual HTS profile along the whole chromosome II. Some regions are repetitive on the both strands (1, 2) and some others are only repetitive on one strand (3, 4). This figure illustrates that virtual HTS profile is able to discriminate repetitiveness according to the strand in the reference genome.
