# Supplementary material for: Sequence Profiling of the Saccharomyces cerevisiae Genome Permits Deconvolution of Unique and Multialigned Reads for Variant Detection
Source: G3 (Bethesda). 2014 Feb 20;4(4):707–15. doi: 10.1534/g3.113.009464 (PMC4059241; doi:10.1534/g3.113.009464)
Supplement: Supporting Information [file supp_g3.113.009464_FigureS4.pdf]

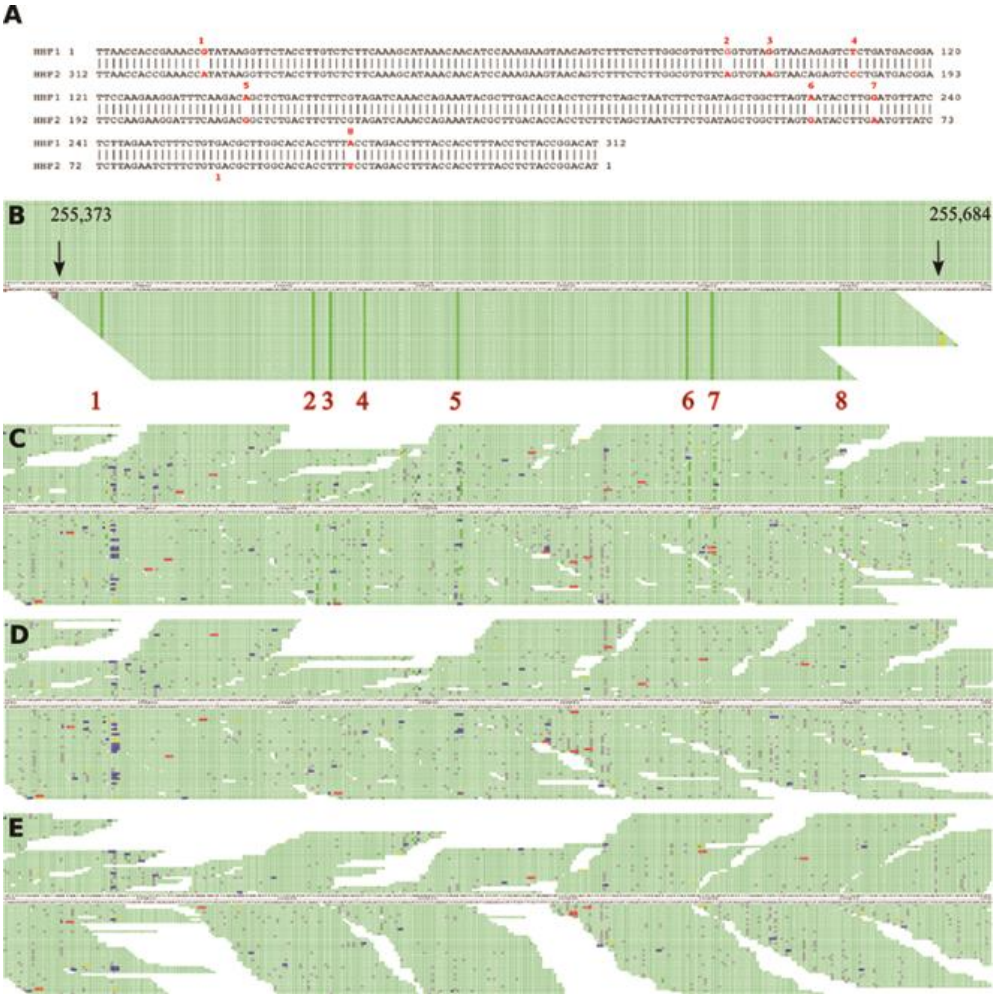

Figure S4 Application of the g-deNoise filtering on the duplicated histone H4 *HHF1* multi-aligned regions. (A) Pairwise alignments of *HHF1* (chr. II: 255,373-255,684) and *HHF2* (chr. XIV: 576,727-577,038). The two genes, *HHF1* and *HHF2,* exhibit eight base changes (numbered). (B) Multi-aligned simulated reads. As *HHF1* and *HHF2* are inverted copies, reads generated on *HHF1* are aligned on the forward strand and reads generated on *HHF2* are aligned on the reverse strand. (C) Multi-aligned reads in experimental data. Light green, yellow and red positions suggest one, two and three base changes respectively. Grey and blue positions: sequencing errors. (D) Alignments of experimental data after discarding alignments consistent with an intra SNV, and (E) remaining unique-alignments after discarding multi-alignments in (D). Here, with 50 nt-reads, we observe that the whole gene sequence is still covered by alignments and is therefore now reduced into a M_u region prone to robust SNP polymorphism detection.
