# Supplementary material for: Sequence Profiling of the Saccharomyces cerevisiae Genome Permits Deconvolution of Unique and Multialigned Reads for Variant Detection
Source: G3 (Bethesda). 2014 Feb 20;4(4):707–15. doi: 10.1534/g3.113.009464 (PMC4059241; doi:10.1534/g3.113.009464)
Supplement: Supporting Information [file supp_g3.113.009464_FigureS5.pdf]

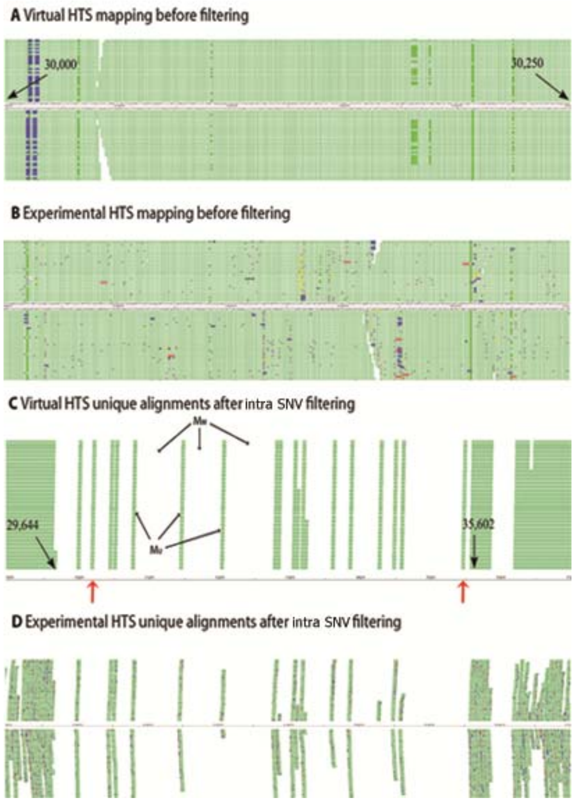

Figure S5 Visualization of reads mapping on the M regions of *YBLWTy2-1*. Alignments without filtering are represented along the 30,000-30,250 sequence of *YBLWTy2-1* (chr. II: 29,644-35,602) containing the TYA Gag gene, obtained in (A) the virtual HTS profile and in (B) the experimental HTS data. The (C) and (D) panels represent the M_U regions along the *YBLWTy2-1* (~6kb) sequence in the virtual HTS profile and corresponding HTS experimental data, respectively. Note that two M_U regions peaks, indicated by red arrows in virtual HTS profile (C), are missing in the HTS experimental data (D). This indicates the loss of specific polymorphism in the experimental sample, probably a loss or gain of an intra SNVs.
