# Supplementary material for: Sequence Profiling of the Saccharomyces cerevisiae Genome Permits Deconvolution of Unique and Multialigned Reads for Variant Detection
Source: G3 (Bethesda). 2014 Feb 20;4(4):707–15. doi: 10.1534/g3.113.009464 (PMC4059241; doi:10.1534/g3.113.009464)
Supplement: Supporting Information [file supp_g3.113.009464_TableS1.pdf]

**Table S1 Functional annotation of M regions along chromosome II**

| #peak | M region start | M region stop | Systematic name | Standard name |
|---|---|---|---|---|
| **1** | 1 | 5856 | TEL02L | |
| **1** | | | TEL02L-YP | |
| **1** | | | YBL113C | |
| **1** | | | YBL113W-A | |
| **1** | | | TEL02L-YP_repeat_region | |
| **1** | | | YBL112C | |
| **1** | | | YBL111C | |
| **1** | | | YBL109W | |
| **1** | | | TEL02L-XR | |
| **1** | 5905 | 8310 | YBL109W | |
| **1** | | | TEL02L-XR | |
| **1** | | | TEL02L-XC | |
| **1** | | | YBL108C-A | *PAU9* |
| **1** | | | YBL108W | |
| **1** | 8328 | 8420 | YBL108W | |
| **1** | 8864 | 8915 | YBLWdelta2 | |
| **1** | 8944 | 9012 | YBLWdelta2 | |
| **1** | 9080 | 9434 | YBLWdelta3 | |
| **1** | 9569 | 9684 | tL(UAA)B1 | |
| **2** | 29623 | 35611 | YBLWdelta4 | |
| **2** | | | YBLWty2-1 | |
| **2** | | | YBLWdelta5 | |
| **2** | | | YBWdelta6 | |
| **2** | 35838 | 36229 | YBLCtau1 | |
| **2** | 36385 | 36496 | tF(GAA)B | |
| **3** | 59834 | 60099 | YBL087C | *RPL23A* |
| **3** | 60101 | 60192 | YBL087C | *RPL23A* |
| **4** | 68267 | 68356 | YBL084C | *CDC27* |
| **5** | 72337 | 72410 | YBL081C | |
| **6** | 88512 | 89126 | YBL072C | *RPS8A* |
| **6** | 92826 | 92875 | YBL068W | *PRS4* |
| **7** | 166365 | 166436 | YBL029W | |
| **7** | 168808 | 168889 | YBL027W | *RPL19B* |
| **7** | 168927 | 169201 | YBL027W | *RPL19B* |
| **7** | 169209 | 169379 | YBL027W | *RPL19B* |
| **7** | 175875 | 175929 | YBL023C | *MCM2* |
| **8** | 187312 | 187368 | YBL017C | *PEP1* |
| **8** | 187673 | 187781 | YBL017C | *PEP1* |
| **8** | 187991 | 188054 | YBL017C | *PEP1* |
| **8** | 188351 | 188418 | YBL017C | *PEP1* |
| **8** | 188477 | 188590 | YBL017C | *PEP1* |
| **8** | 188592 | 188742 | YBL017C | *PEP1* |
| **8** | 188877 | 188928 | YBL017C | *PEP1* |
| **8** | 188930 | 188994 | YBL017C | *PEP1* |
| **8** | 189002 | 189051 | YBL017C | *PEP1* |
| **9** | 197488 | 197583 | tI(AAU)B | |
| **10** | 197616 | 198069 | tG(GCC)B | |
| **10** | | | YBLWsigma1 | |
| **11** | 205746 | 205813 | YBL011W | *SCT1* |
| **12** | 221028 | 226963 | YBLWdelta8 | |
| **12** | | | YBLWdelta9 | |
| **12** | | | YBLWTy1-1 | |
| **12** | | | YBLWdelta10 | |
| **12** | 227062 | 227177 | tS(AGA)B | |

| | | | | |
|---|---|---|---|---|
| **13** | 235403 | 235810 | YBL003C | *HTA2* |
| **13** | 236618 | 236763 | YBL002W | *HTB2* |
| **13** | 255367 | 255688 | ARS209 | *ARS209* |
| **13** | | | YBR009C | *HHF1* |
| **13** | 256374 | 256624 | YBR010W | *HHT1* |
| **13** | 256683 | 256737 | YBR010W | *HHT1* |
| **13** | 258689 | 258810 | YBRCdelta11 | |
| **13** | 258859 | 258926 | YBRCdelta11 | |
| **13** | 259564 | 263149 | YBR012C | |
| **13** | | | YBRWdelta12 | |
| **13** | | | YBRWTy1-2 | |
| **13** | 263164 | 265504 | YBRWTy1-2 | |
| **13** | 266172 | 266256 | YBRCdelta14 | |
| **13** | 266367 | 266465 | tT(AGU)B | |
| **13** | 270424 | 270488 | YBR016W | |
| **14** | 300159 | 301247 | YBR031W | *RPL4A* |
| **15** | 326780 | 326872 | tV(UAC)B | |
| **15** | 327163 | 327352 | YBRWdelta15 | |
| **16** | 332826 | 332880 | YBR048W | *RPS11B* |
| **16** | 333383 | 333810 | YBR048W | *RPS11B* |
| **16** | 343123 | 343181 | YBR054W | *YRO2* |
| **16** | 343564 | 343616 | YBR054W | *YRO2* |
| **17** | 347591 | 347704 | / | |
| **17** | 350815 | 350906 | tQ(UUG)B | |
| **17** | 353582 | 353664 | / | |
| **17** | 372325 | 372392 | YBR067C | *TIP1* |
| **18** | 405845 | 406059 | tR(UCU)B | |
| **18** | | | tD(GUC)B | |
| **19** | 414180 | 414350 | YBR084C-A | *RPL19A* |
| **19** | 414358 | 414632 | YBR084C-A | *RPL19A* |
| **19** | 414670 | 414751 | YBR084C-A | *RPL19A* |
| **20** | 427755 | 428109 | YBR092C | *PHO3* |
| **20** | 428336 | 428399 | YBR092C | *PHO3* |
| **20** | 428485 | 428576 | YBR092C | *PHO3* |
| **20** | 428671 | 428830 | YBR092C | *PHO3* |
| **20** | 428883 | 428984 | YBR092C | *PHO3* |
| **20** | 429576 | 429959 | YBR093C | *PHO5* |
| **20** | 430186 | 430249 | YBR093C | *PHO5* |
| **20** | 430335 | 430426 | YBR093C | *PHO5* |
| **20** | 430521 | 430680 | YBR093C | *PHO5* |
| **20** | 430698 | 430834 | YBR093C | *PHO5* |
| **20** | 430881 | 430936 | YBR093C | *PHO5* |
| **21** | 463997 | 464290 | YBR112C | *CYC8* |
| **22** | 477659 | 479049 | YBR118W | *TEF2* |
| **23** | 541431 | 541526 | YBR150C | *TBS1* |
| **23** | 541577 | 541639 | YBR150C | *TBS1* |
| **24** | 591706 | 592422 | YBR181C | *RPS6B* |
| **25** | 604937 | 605061 | YBR189W | *RPS9B* |
| **25** | 605069 | 605152 | YBR189W | *RPS9B* |
| **25** | 605195 | 605451 | YBR189W | *RPS9B* |
| **25** | 606653 | 606731 | YBR191W | *RPL21A* |
| **25** | 606734 | 606918 | YBR191W | *RPL21A* |
| **25** | 606920 | 607131 | YBR191W | *RPL21A* |
| **25** | 627307 | 627364 | YBR202W | *MCM7* |
| **26** | 642988 | 643084 | tC(GCA)B | |
| **26** | 643470 | 643865 | YBRCtau2 | |
| **26** | | | YBRWdelta18 | |
| **26** | 645147 | 645250 | tE(UUC)B | |
| **26** | 646075 | 646133 | / | |

| | | | | |
|---|---|---|---|---|
| **27** | 659261 | 659328 | YBR218C | *PYC2* |
| **27** | 659576 | 659630 | YBR218C | *PYC2* |
| **27** | 659645 | 659730 | YBR218C | *PYC2* |
| **27** | 659774 | 659853 | YBR218C | *PYC2* |
| **27** | 660212 | 660594 | YBR218C | *PYC2* |
| **27** | 660610 | 660759 | YBR218C | *PYC2* |
| **27** | 660791 | 660948 | YBR218C | *PYC2* |
| **27** | 661274 | 661362 | YBR218C | *PYC2* |
| **27** | 661406 | 661455 | YBR218C | *PYC2* |
| **27** | 661460 | 661757 | YBR218C | *PYC2* |
| **27** | 661835 | 661965 | YBR218C | *PYC2* |
| **27** | 701990 | 702056 | / | |
| **27** | 780350 | 780468 | YBR289W | *SNF5* |
| **28** | 801690 | 801919 | YBR297W | *MAL33* |
| **29** | 804863 | 808394 | YBR298C-A | |
| **29** | | | YBR299W | *MAL32* |
| **29** | 808396 | 813178 | YBR300C | |
| **29** | | | YBR301W | *PAU24* |
| **29** | | | YBR302C | *COS2* |
