# Supplementary material for: Sequence Profiling of the Saccharomyces cerevisiae Genome Permits Deconvolution of Unique and Multialigned Reads for Variant Detection
Source: G3 (Bethesda). 2014 Feb 20;4(4):707–15. doi: 10.1534/g3.113.009464 (PMC4059241; doi:10.1534/g3.113.009464)
Supplement: Supporting Information [file supp_g3.113.009464_TableS2.pdf]

Table S2   Constitutive SNPs found in the U regions in the wild-type mutation accumulation lines

Line	Chrom.	Position	ORF	Gene	Ref.	Variant	Aa change	Sequence Ontology term
wt_ABCE100	chrII	15 101	YBL105C	*PKC1*	T	G	I866L	missense_variant
wt_ABCE100	chrIII	143 131	Intergenic	-	T	C	-	intergenic_variant
wt_ABCE100	chrIII	162 361	Intergenic	-	T	C	-	intergenic_variant
wt_ABCE100	chrIII	162 640	YCR024C-B	YCR024C-B	T	G	S76R	missense_variant
wt_ABCE100	chrIII	162 694	YCR024C-B	YCR024C-B	G	A	synonymous	synonymous_variant
wt_ABCE100	chrIII	163 059	YCR024C-A	*PMP1*	T	C	synonymous	synonymous_variant
wt_ABCE100	chrV	48 384	Intergenic	-	T	C	-	intergenic_variant
wt_ABCE100	chrV	154 531	YER001W	*MNN1*	T	A	S338T	missense_variant
wt_ABCE100	chrV	352 394	YER096W	*SHC1*	A	G	K233E	missense_variant
wt_ABCE100	chrV	517 529	Intergenic	-	T	C	-	intergenic_variant
wt_ABCE100	chrXIII	448 333	Intergenic	-	G	A	-	intergenic_variant
wt_ABCE100	chrXIII	680 936	YMR207C	*HFA1*	T	C	K877E	missense_variant
wt_ABCE100	chrXIII	680 940	YMR207C	*HFA1*	C	T	synonymous	synonymous_variant
