# Supplementary material for: Sequence Profiling of the Saccharomyces cerevisiae Genome Permits Deconvolution of Unique and Multialigned Reads for Variant Detection
Source: G3 (Bethesda). 2014 Feb 20;4(4):707–15. doi: 10.1534/g3.113.009464 (PMC4059241; doi:10.1534/g3.113.009464)
Supplement: Supporting Information [file supp_g3.113.009464_TableS3.pdf]

Table S3 Acquired SNPs found in the U regions in the wild-type mutation accumulation lines

Line	Chrom.	Position	ORF	Gene	Ref.	Variant	Aa change	Sequence Ontology term
wt_A100	chrII	468 275	YBR114W	*RAD16*	C	T	T343M	missense_variant
wt_B100	chrIII	1 989	YCL076W	YCL076W	G	A	G200S	missense_variant
wt_B100	chrIV	191 155	YDL147W	*RPN5*	C	A	Q78K	missense_variant
wt_A100	chrIV	573 098	YDR060W	*MAK21*	A	T	D817V	missense_variant
wt_B100	chrIV	738 528	YDR141C	*DOP1*	A	C	I490M	missense_variant
wt_A100	chrIV	866 198	YDR207C	*UME6*	C	T	R442Q	missense_variant
wt_E100	chrIV	1 171 524	YDR348C	*PAL1*	G	A	P101L	missense_variant
wt_E100	chrIV	1 452 784	YDR501W	*PLM2*	G	T	E478STOP	stop_gained
wt_B100	chrIV	1 486 605	YDR523C	*SPS1*	G	T	A145D	missense_variant
wt_C100	chrV	234 495	YER041W	*YEN1*	T	C	S679P	missense_variant
wt_C100	chrV	312 202	Intergenic	-	A	G	-	intergenic_variant
wt_E100	chrV	404 934	YER123W	*YCK3*	A	G	Q41R	missense_variant
wt_C100	chrVII	313 763	YGL100W	*SEH1*	G	T	R177I	missense_variant
wt_E100	chrVII	376 132	YGL067W	*NPY1*	A	T	H11L	missense_variant
wt_C100	chrVII	1 041 377	YGR274C	*TAF1*	G	T	synonymous	synonymous_variant
wt_B100	chrVIII	137 015	YHR016C	*YSC84*	G	A	P425S	missense_variant
wt_A100	chrVIII	275 555	YHR084W	*STE12*	A	G	Y461C	missense_variant
wt_A100	chrVIII	368 794	YHR132C	*ECM14*	A	G	L334S	missense_variant
wt_A100	chrVIII	390 799	YHR146W	*CRP1*	C	T	T167I	missense_variant
wt_A100	chrX	618 251	YJR101W	*RSM26*	G	A	synonymous	synonymous_variant
wt_A100	chrXI	8 145	Intergenic	-	G	C	-	intergenic_variant
wt_A100	chrXI	291 061	Intergenic	-	C	T	-	intergenic_variant
wt_B100	chrXI	480 315	YKR021W	*ALY1*	A	C	E361A	missense_variant
wt_E100	chrXII	197 723	YLR027C	*AAT2*	T	A	K121N	missense_variant
wt_B100	chrXII	318 957	YLR089C	*ALT1*	C	A	Q353H	missense_variant
wt_C100	chrXII	357 349	YLR106C	*MDN1*	G	A	synonymous	synonymous_variant
wt_E100	chrXII	360 374	YLR106C	*MDN1*	T	G	Y1122S	missense_variant
wt_B100	chrXII	837 516	Intergenic	-	C	T	-	intergenic_variant
wt_E100	chrXII	935 171	YLR409C	*UTP21*	G	C	S688W	missense_variant
wt_E100	chrXII	1 034 119	YLR450W	*HMG2*	T	G	F498C	missense_variant
wt_B100	chrXIII	470 741	YMR102C	YMR102C	T	A	S538C	missense_variant
wt_A100	chrXIII	912 702	Intergenic	-	G	T	-	intergenic_variant
wt_B100	chrXIV	275 036	YNL193W	YNL193W	G	A	synonymous	synonymous_variant
wt_C100	chrXIV	752 345	YNR065C	YNR065C	G	C	S452R	missense_variant
wt_A100	chrXV	406 610	Intergenic	-	T	C	-	intergenic_variant
wt_C100	chrXV	594 444	Intergenic	-	A	T	-	intergenic_variant
wt_C100	chrXVI	120 124	Intergenic	-	A	T	-	intergenic_variant
wt_A100	chrXVI	307 821	YPL128C	*TBF1*	G	A	T134I	missense_variant
wt_A100	chrXVI	510 123	Intergenic	-	G	T	-	intergenic_variant
